# Phosphorylation of the LIR Domain of SCOC Modulates ATG8 Binding Affinity and Specificity

**DOI:** 10.1016/j.jmb.2021.166987

**Published:** 2021-06-25

**Authors:** Martina Wirth, Stephane Mouilleron, Wenxin Zhang, Eva Sjøttem, Yakubu Princely Abudu, Ashish Jain, Hallvard Lauritz Olsvik, Jack-Ansgar Bruun, Minoo Razi, Harold B.J. Jefferies, Rebecca Lee, Dhira Joshi, Nicola O'Reilly, Terje Johansen, Sharon A. Tooze

**Affiliations:** 1 Molecular Cell Biology of Autophagy, The Francis Crick Institute, 1 Midland Road, London NW1 1AT, UK; 2 Structural Biology Science Technology Platforms, The Francis Crick Institute, 1 Midland Road, London NW1 1AT, UK; 3 Peptide Chemistry Science Technology Platforms, The Francis Crick Institute, 1 Midland Road, London NW1 1AT, UK; 4 Molecular Cancer Research Group, Department of Medical Biology, University of Tromsø - The Arctic University of Norway, 9037 Tromsø, Norway; 5 Department of Cell and Chemical Biology, Leiden University Medical Center, Einthovenweg 20, 2333 ZC Leiden, the Netherlands‡; 6 Department of Molecular Cell Biology, Centre for Cancer Biomedicine, Institute for Cancer Research, The Norwegian Radium Hospital, Montebello, N-0379 Oslo, Norway‡

**Keywords:** autophagy, LIR motif, phosphorylation, bio-layer interferometry, crystal structure

## Abstract

Autophagy is a highly conserved degradative pathway, essential for cellular homeostasis and implicated in diseases including cancer and neurodegeneration. Autophagy-related 8 (ATG8) proteins play a central role in autophagosome formation and selective delivery of cytoplasmic cargo to lysosomes by recruiting autophagy adaptors and receptors. The LC3-interacting region (LIR) docking site (LDS) of ATG8 proteins binds to LIR motifs present in autophagy adaptors and receptors. LIR-ATG8 interactions can be highly selective for specific mammalian ATG8 family members (LC3A-C, GABARAP, and GABARAPL1-2) and how this specificity is generated and regulated is incompletely understood.

We have identified a LIR motif in the Golgi protein SCOC (short coiled-coil protein) exhibiting strong binding to GABARAP, GABARAPL1, LC3A and LC3C. The residues within and surrounding the core LIR motif of the SCOC LIR domain were phosphorylated by autophagy-related kinases (ULK1-3, TBK1) increasing specifically LC3 family binding. More distant flanking residues also contributed to ATG8 binding. Loss of these residues was compensated by phosphorylation of serine residues immediately adjacent to the core LIR motif, indicating that the interactions of the flanking LIR regions with the LDS are important and highly dynamic.

Our comprehensive structural, biophysical and biochemical analyses support and provide novel mechanistic insights into how phosphorylation of LIR domain residues regulates the affinity and binding specificity of ATG8 proteins towards autophagy adaptors and receptors.

## Highlights

• Autophagic degradation of cellular material relies on LIR-ATG8 interactions.
• Regulation of LIR-ATG8 interactions by phosphorylation is incompletely understood.
• The Golgi protein SCOC binds to ATG8 proteins through a functional LIR domain.
• SCOC LIR phosphorylation by ULK1-3, and TBK1 increases specifically LC3 binding.
• New LIR phospho -regulation critical for ATG8 binding affinity and specificity.

## Graphical abstract

**Figure:**
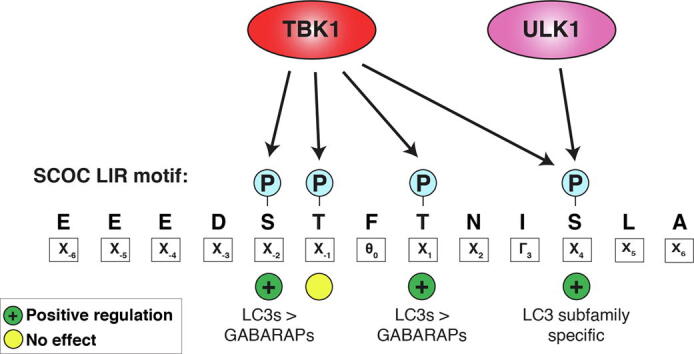

## Introduction

Autophagy is a highly conserved recycling and stress survival pathway ensuring cellular health and homeostasis.^1^ During starvation, autophagy restores intracellular nutrients and molecular building blocks by sequestering and transferring cytosolic material in autophagosomes to lysosomes for degradation. Moreover, autophagy selectively removes toxic macromolecules, damaged organelles, and intracellular pathogens and therefore has been implicated in pathologies such as neurodegeneration, cancer, and infection.^2^, ^3^, ^4^, ^5^

The molecular machinery driving autophagosome formation comprises autophagy-related (ATG) proteins which are highly conserved from yeast to human. In mammals, autophagy is initiated by the UNC-51 like kinase (ULK) complex, which phosphorylates and activates the class III ATG14-Beclin1-VPS34-p150 phosphatidylinositol 3-phosphate (PI3P) kinase complex I.^6^, ^7^ This results in production of PI3P at autophagosome formation sites (omegasomes) on the endoplasmic reticulum (ER) and recruitment of PI3P-binding effectors, such as DFCP1 and WIPI proteins.^8^ WIPI2b further recruits the ATG12-5-16L1 complex^9^; which mediates conjugation of ATG8 proteins to phosphatidylethanolamine in the autophagic membrane.^10^, ^11^

Whereas yeast only has one ATG8 protein, mammalian cells have two ATG8 protein subfamilies, namely LC3s (LC3A, LC3B, LC3C) and GABARAPs (GABARAP, GABARAP-L1, GABARAP-L2),^12^ functioning in autophagosome formation and fusion with the lysosome.^13^, ^14^, ^15^, ^16^ During selective autophagy, ATG8 proteins mediate cargo recruitment by directly interacting with autophagy receptors, such as p62/SQSTM1.^17^ Moreover, interactions of ATG8 proteins with autophagy adaptors which are not degraded by autophagy regulate autophagosome formation (e.g. ULK1, PI3K class III complex, ATG2 and ATG4),^18^, ^19^, ^20^, ^21^, ^22^ fusion with the lysosome (e.g. PLEKHM1),^23^ lysosome or autolysosome biogenesis (e.g. STX16 and STX17),^24^ or autophagosome transport (e.g. FYCO1).^25^

Interaction with ATG8 proteins typically occurs via an ATG8-interacting motif (AIM), also known as LC3-interacting region (LIR) motif.^5^, ^26^, ^27^, ^28^ The consensus sequence of the canonical LIR motif is a small Θ_0_-X_1_-X_2_-Γ_3_ motif with Θ representing an aromatic residue (W/F/Y), Γ an aliphatic residue (L/V/I), and X any amino acid (aa). The side chains of the aromatic and aliphatic residues fit into hydrophobic pocket 1 (HP1) and 2 (HP2) of the ATG8 LIR docking site (LDS), respectively. Acidic and phosphorylated residues, often present in the N-terminal region directly preceding the core LIR motif, stabilize ATG8 binding through electrostatic interactions.^26^, ^27^, ^28^

Many LIR-containing proteins interact with ATG8 proteins in a highly selective manner, suggesting functional differences between LC3 and GABARAP subfamily proteins.^5^, ^13^, ^14^, ^15^, ^28^ Rogov et al. defined a GABARAP interaction motif (GIM), Θ_0_-[V/I]_1_-X_2_-Γ_3_, and showed that valine or isoleucine in position X_1_ promotes GABARAP binding.^29^ We recently showed that the binding preference of LIR motifs to GABARAPs is also regulated by the X_2_ residue within the core LIR motif as well as residues in the adjacent C-terminal region (positions X_4_-X_10_).^30^ ATG8-subfamily binding specificity is also determined by the residues of the LIR-docking site (LDS). In line with these findings, a recent study on plant ATG8 proteins revealed that an amino acid polymorphism in the N-terminal beta-strand comprising HP1 of the LDS determines the binding specificity of two potato ATG8 isoforms and contributes to their functional specialization.^31^

In addition, phosphorylation has been shown to modulate ATG8 binding specificity.^20^, ^32^, ^33^, ^34^, ^35^, ^36^, ^37^, ^38^ The LIR motif of the autophagy receptor optineurin (OPTN)^32^ interacts strongly with GABARAP and weakly with LC3B. Phosphorylation of OPTN serine S177 at position X_−1_, (the residue directly preceding the core LIR motif) by TANK-binding kinase1 (TBK1) activates LC3B binding and promotes selective autophagy of cytosolic Salmonella.^32^ Phosphorylation of residues N-terminal to the core LIR motif also positively regulates GABARAP binding to the VPS34 and Beclin1 LIR motifs.^20^ Conversely; phosphorylation of S13 (position X_−5_)^37^ and Y18; the aromatic core LIR motif residue (position Θ_0_)^35^; of the mitophagy receptor FUNDC1 prevent LC3 binding and inhibit mitophagy. Further analyses are required to fully understand the role of phosphorylation, the molecular determinants and constraints that confer and regulate selective ATG8 binding.

A genome wide siRNA screen identified the short coiled-coil protein (SCOC) as a novel positive regulator of starvation induced autophagy.^39^ SCOC is a small Golgi protein implicated in Golgi transport through interacting with ADP-ribosylation factor-like 1 (ARL1) via its coiled-coil domain.^40^ The SCOC coiled-coil domain also mediates a highly conserved interaction with the kinesin1-adaptor protein fasciculation and elongation protein zeta 1 (FEZ1)^39^, ^41^, ^42^, ^43^ and both proteins are required for axonal growth; transport and normal presynaptic organization.^41^, ^44^, ^45^ Interestingly; the Drosophila ULK1 homolog UNC-51 regulates axonal transport by binding and phosphorylating FEZ1 (UNC-76).^46^ The interaction between ULK1 and FEZ1 is also conserved in mammals and might be modulated by SCOC.^39^

To further understand the role of SCOC in autophagy, we identified a functional LIR motif in the unstructured region N-terminal of the coiled-coil domain of SCOC. The SCOC LIR motif exhibits a high binding preference for GABARAP, GABARAPL1 as well as LC3A and LC3C. The molecular details mediating SCOC LIR-ATG8 binding were investigated by X-ray crystallography and bio-layer interferometry (BLI) affinity measurements. Our data demonstrates a critical contribution of the flanking LIR domain residues, which are up to eight amino acid residues distant from the SCOC core LIR motif, to ATG8 protein binding. Moreover, we show that ULK kinases and TBK1 can phosphorylate SCOC serine/threonine residues (T15/S18^SCOC^) within and C-terminal to the core LIR motif to strongly increase the affinity to LC3 subfamily proteins. These data support and extend the notion of key phosphorylation sites that can regulate ATG8 binding affinity and specificity of LIR motifs.

## Results

### SCOC interacts with mammalian ATG8 proteins through a N-terminal LIR domain

**Figure 1:**
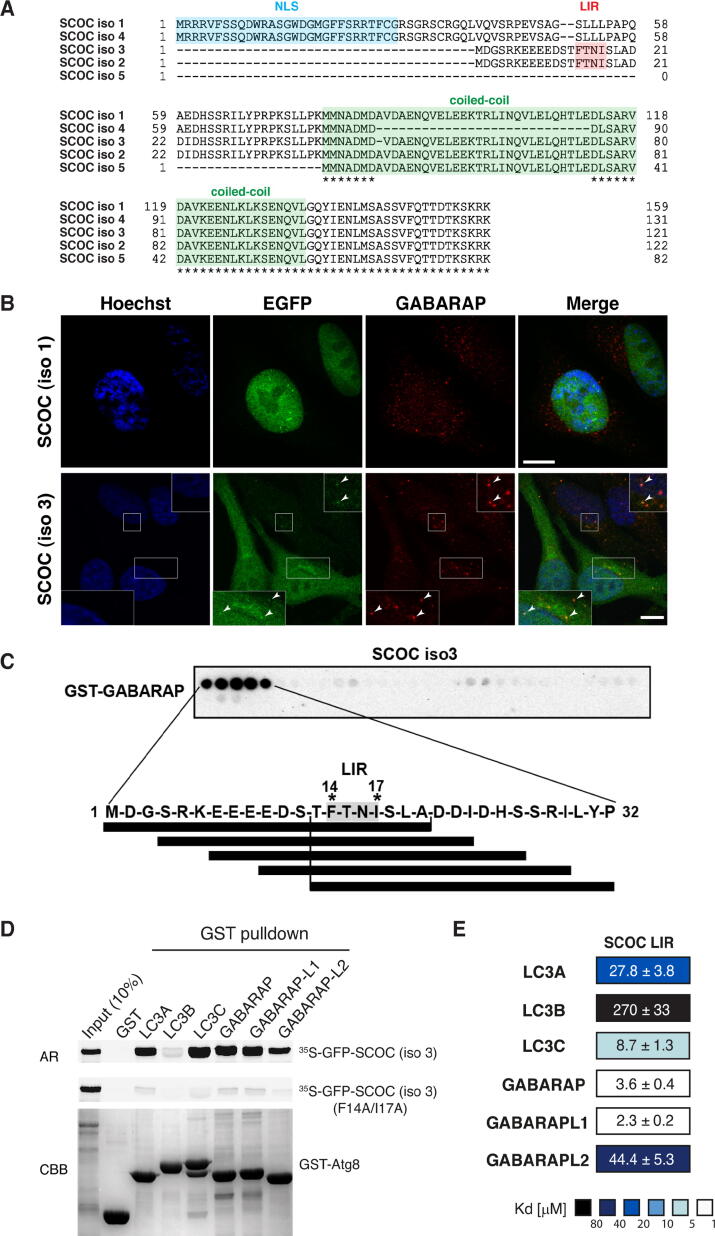
SCOC interacts with mammalian ATG8 proteins through a N-terminal LIR domain. A. Sequence alignment of SCOC isoform 1 (Q9UIL1), SCOC isoform 4 (Q9UIL1-4), SCOC isoform 3 (Q9UIL1-3), SCOC isoform 2 (Q9UIL1-2), SCOC isoform 5 (A0A0C4DGB0/protein accession AAK01707) using the Clustal Omega program of the UniProt database (www.uniprot.org). The highly conserved coiled-coil domain is highlighted in green. The LIR motif and the nuclear localisation signal sequence are indicated in red and blue, respectively. Residues conserved in all five isoforms are highlighted by asterisks (*) B. HeLa Flp-In T-Rex cells stably expressing EGFP-SCOC (isoform 1) or EGFP-SCOC (isoform 3) starved for 2 h in EBSS, fixed and labelled with anti-GABARAP and Hoechst for confocal microscopy. Expression of EGFP-SCOC constructs was induced with 0.5 μg/ml tetracycline overnight. Scale bars represent 10 μM. White boxes indicate position of insets and arrows indicate EGFP-SCOC (isoform 3)-GABARAP colocalization. C. Peptide array of 20-mer peptides covering full-length SCOC isoform 3 (each peptide shifted 3 amino acids relative to the previous) was incubated with GST-GABARAP and immunoblotted with anti-GST. The amino acid sequence for the GABARAP interacting peptides is shown with the interacting peptides depicted as black lines. D. GST pulldown assay of *in vitro* translated and [^35^S]methionine labelled wild-type (WT) and LIR mutant (F14A/I17A) GFP-SCOC (isoform 3) proteins binding to GST-ATG8 proteins. Autoradiograph (AR, upper panels) and representative coomassie stained immobilized GST fusion proteins (CBB, bottom panel) are shown. E. Affinities (K_d_ values) of SCOC (WT) LIR domain peptide to human ATG8 proteins determined by bio-layer interferometry (BLI). Color code indicates fold changes relative to GABARAP (data are average Kd values ± standard error calculated from non-linear regression curve fits, n = 2).

SCOC is widely expressed in human tissues^40^, ^41^ and due to alternative splicing multiple isoforms have been reported. All SCOC isoforms are highly conserved in the C-terminal region comprising a coiled-coil domain; but vary in the N-terminal region (Figure 1A). To elucidate whether the diverse N-terminal regions confer functional differences we employed the T-REX^TM^ HeLa cell line system to generate stable inducible HeLa cell lines expressing EGFP-tagged SCOC isoform 1 and 3 (Figure 1B). SCOC isoform 1 localizes primarily to the nucleus in interphase cells and possesses a potential nuclear localization signal (NLS) located in the N-terminal region (aa 1–29) (Figure 1A, B, predicted with cNLS Mapper tool.^47^ During cell division, a pronounced localization of SCOC isoform1 to the mitotic spindle (labelled by tubulin) was detected in prophase, metaphase, and anaphase cells (Supplementary Figure 1A). In contrast, SCOC isoform 3 distributed to both the nucleus and cytosol (Figure 1B), where it partially colocalized with the trans-Golgi network (TGN) marker TGN46 (Supplementary Figure 1B). Notably, the localisation of EGFP-SCOC isoform 3 to the Golgi and trans-Golgi network was less prominent than endogenous SCOC reported by immunofluorescence staining using an antibody recognising the conserved coiled-coil region (Figure 1A).^39^, ^40^ Endogenous SCOC partially colocalises with endogenous LC3 in EBSS-starved cells.^39^ In response to EBSS starvation; we detected partial colocalization of SCOC isoform 3-positive puncta with endogenous GABARAP (Figure 1B). Due to potential cross-reactivity of the anti-GABARAP antibody (Abgent) with other GABARAP subfamily proteins^48^; SCOC isoform 3 might also partially colocalize with GABARAPL1 and GABARAPL2.

Bioinformatic analyses (iLIR^49^ identified a putative LIR motif (aa 13-FTNI-aa 18) in the non-conserved N-terminal region of both SCOC isoform 2 and 3 (varying by a single aa (alanine) at residue 48) (Figure 1A). This LIR motif was confirmed by probing a peptide array of 20-mer peptides, with each peptide shifted 3 amino acids C-terminally to cover full-length SCOC isoform 3, with GST-GABARAP (Figure 1C). In GST-pull down experiments SCOC isoform 3 interacted strongly with LC3A, LC3C, GABARAP and GABARAPL1, much weaker with GABARAPL2 and very little with LC3B (Figure 1D). Mutation of the LIR motif residues F14 and I17 to alanine (F14A/I17A) reduced the interaction with all ATG8 proteins and confirmed that this LIR motif is functional. However, these mutations did not abrogate colocalization of SCOC isoform 3 (F14A/I17A) with TGN46 and GABARAP (Supplementary Figure 1B) suggesting that the LIR motif is not essential for SCOC localization to the trans-Golgi network and GABARAP-positive puncta and this localization might occur through binding other proteins, such as Arl1 and FEZ1,^39^, ^40^ or dimerization with endogenous wild type SCOC.^50^

In line with the GST pull down experiments, biolayer-interferometry (BLI) affinity measurements showed that the SCOC LIR binds strongest to GABARAP (3.6 µM) and GABARAPL1 (2.3 µM), followed by LC3C (8.7 µM) and LC3A (27.8 µM), and weakest to GABARAPL2 (44.4 µM), and LC3B (270 µM) (Figure 1E, Supplementary Figure 2A). The binding affinities of the SCOC LIR motif, ranging from 2-300 µM are similar to Kd values reported for the autophagy adaptors PCM1^30^ and the PI3K (class III) complex members, Beclin1, VPS34 and ATG14.^20^

To determine whether SCOC is functioning as an autophagy receptor and degraded by autophagy, we induced expression of EGFP-SCOC (isoform 1 and 3) in HeLa cells followed by 7 h EBSS starvation in the presence and absence of lysosomal or proteasomal inhibitors. Inhibition of proteasomal degradation using MG132 or epoxymycin, increased SCOC isoform 1 and 3 protein levels, whereas treatment with BafilomycinA1 (BafA1), which inhibits lysosomal degradation, did not have any effect in both fed and starved cells (Supplementary Figure 2B, 2C). Thus, both SCOC isoform1 and 3 are not turned over by autophagy, but by the proteasome. In summary, SCOC isoform 3 shows a high binding affinity to GABARAP, GABARAPL1, and LC3C and potentially is a novel autophagy adaptor protein.

### Molecular determinants mediating SCOC LIR-GABARAP complex formation

**Figure 2:**
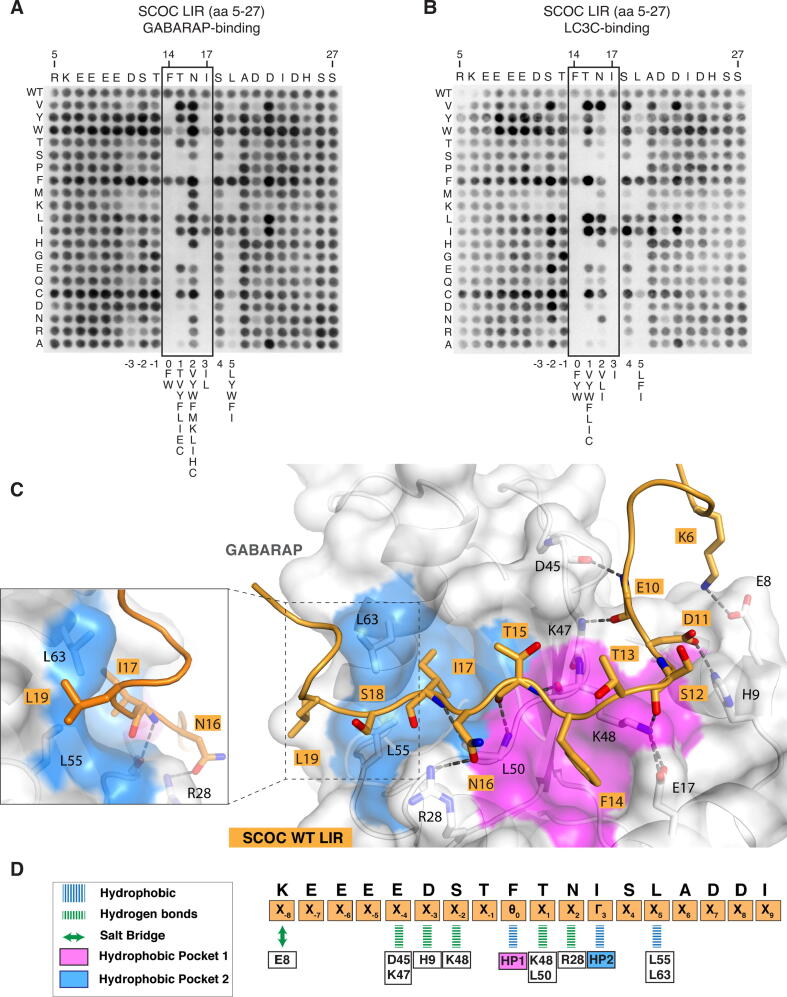
Molecular determinants mediating SCOC LIR-GABARAP complex formation. A. and B. Mutational peptide array of 23-mer SCOC peptides covering the LIR motif incubated with GST-GABARAP (A.) or GST-LC3C (B.) and immunoblotted with anti-GST. Each amino acid position was substituted for every other amino acid. C. Structure of the SCOC LIR domain (aa 6–23) bound to GABARAP. The SCOC LIR sequence is shown in orange cartoon with interacting residues shown as sticks. GABARAP is displayed in white cartoon and transparent surface with hydrophobic pocket 1 and 2 coloured in pink and blue surfaces, respectively. The box with dashed lines indicates location of close-up view showing hydrophobic interactions of L19^SCOC^ with the edge of HP2 (L55/L63^GAB^). D. Schematic overview of the interactions observed in the structure of GABARAP bound to the SCOC LIR domain. Orange boxes below the SCOC sequence display the residue position within the LIR domain. GABARAP residues are boxed and shown in black. Blue lines indicate hydrophobic interactions, green lines hydrogen bonds, and green double arrow salt bridges. Pink and blue boxes indicate canonical LIR motif interactions (hydrophobic contacts) with HP1 and HP2.

The SCOC LIR motif and flanking regions were further characterised using mutational peptide array scans. Every position within the 23-mer SCOC LIR peptide was mutated to all alternative amino acids (aa) and binding of GST-tagged ATG8 proteins was analysed by immunoblotting with an anti-GST antibody. Based on our findings from GST-pull downs (Figure 1D) and BLI affinity measurements (Figure 1E), we focused on strong and medium interacting proteins and selected GST-GABARAP (Figure 2A), GST-LC3C (Figure 2B) and GST-LC3A (Supplementary Figure 2D). The mutational SCOC LIR peptide array scans confirmed a canonical LIR motif, as mutation of both F14 and I17 abrogated binding of all three ATG8 proteins. In line with previous reports,^18^, ^30^, ^51^ GABARAP binding was abolished by glycine or proline substitutions at any position of the core LIR (X_0_-X_3_), whereas LC3C and LC3A binding in addition was abolished by the presence of positively charged amino acids (K and R). Consistent with recent findings,^30^ substitution of N16 by a hydrophobic/aromatic aa (V, L, I, W, Y, F) strongly improved both GABARAP and LC3A/C binding further underscoring the stabilizing effect on ATG8 binding by these aa at the LIR motif position X_2_.^30^ Interestingly, L19 (in position X_5_) was also important for interaction. Only substitutions with the aromatic residues (Y, W, F) and the hydrophobic I gave significant binding, suggesting that residues C-terminal to the core LIR motif also contribute to ATG8 binding. N-terminal to the core LIR motif, substitution of D11 (position X_−3_) slightly reduced LC3A, LC3C and GABARAP binding. Moreover, substitutions of S12 and T13 by D/E improved LC3C (Figure 2B), LC3A (Supplementary Figure 2D) and GABARAP (Figure 2A) binding, further underscoring the positive regulation of LIR motif binding to ATG8 proteins by acidic residues and potential phosphorylation of S/T/Y residues in position X_−1_ and X_−2_.^20^, ^27^, ^30^, ^32^, ^33^, ^34^, ^36^

To further elucidate the molecular determinants mediating interaction of the SCOC LIR motif with GABARAP, we generated a chimera protein consisting of the SCOC^6-23^ LIR domain sequence N-terminally fused to GABARAP with a Gly-Ser linker. We solved the crystal structure of the SCOC^6-23^ LIR domain bound to GABARAP at a resolution of 1.25 Å (Figure 2C, Supplementary Figure 2E, Table 1). The SCOC LIR:GABARAP complex structure revealed a large surface of interactions between SCOC residues 6 to 19 and GABARAP. The SCOC core LIR motif displayed the canonical LIR interactions comprising the hydrophobic residues F14^SCOC^ (Θ_0_) and I17^SCOC^ (Γ_3_) deeply bound to HP1 and HP2, and three hydrogen bonds formed between the main chains of the SCOC LIR residues T15^SCOC^ (X_1_) and I17^SCOC^ (Γ_3_) and the main chains of the GABARAP residues K48^GAB^ and L50^GAB^ (Figure 2C). On top of these canonical LIR interactions additional specific contacts were observed between the SCOC core LIR motif as well as the adjacent N- and C-terminal regions and GABARAP (Figure 2C, D). In the region N-terminal to the SCOC core LIR motif we observed: a salt bridge between K6^SCOC^ (X_−8_) and E8^GAB^, two hydrogen bonds between the backbone of E10^SCOC^ (X_−4_) and the mainchain of D45/K47^GAB^, one hydrogen bond between the sidechain of D11^SCOC^ (X_−3_) and the imidazole ring of H9^GAB^, and another hydrogen bond between the carbonyl of S12^SCOC^ (X_−2_) and K48^GAB^ sidechain. Within the core LIR motif, N16^SCOC^ in position X_2_ was hydrogen bonded with the guanidinium of R28^GAB^. In the C-terminal region, hydrophobic interactions were observed between L19^SCOC^ in position X_5_ and the edge of HP2 (L55/L63^GAB^).

**Table 1:** Data collection and refinement statistics.

	SCOC:GABARAP	SCOC-2pS: GABARAPL1	SCOC-2pT: GABARAPL1
PDB ID	7AA8	7AA7	7AA9
Resolution range	39.92–1.25 (1.29–1.25)	31.92–1.45 (1.47–1.45)	59.94–1.72 (1.78–1.72)
Space group	P 32 2 1	P 1	P 32
Unit cell	52.82 52.82 81.72 90 90 120	28.36 37.17 62.48 81.75 89.60 67.82	90.94 90.94 92.40 90 90 120
Total reflections	289 346 (27 734)	123 975 (6 189)	349 139 (25 323)
Unique reflections	37 098 (3 670)	41 242 (2 051)	90 238 (8 924)
Multiplicity	7.8 (7.6)	3.0 (3.0)	3.9 (2.8)
Completeness (%)	99.84 (99.78)	99.5 (98.9)	99.17 (96.98)
Mean I/sigma(I)	15.85 (1.18)	15.1 (5.0)	7.55 (1.15)
R-merge	0.04 (2.0)	0.09 (0.6)	0.09 (0.8)
R-meas	0.04 (2.1)	0.12 (0.92)	0.11 (0.99)
R-pim	0.01 (0.7)	0.08 (0.60)	0.05 (0.54)
CC1/2	0.99 (0.53)	0.98 (0.46)	0.99 (0.57)
Reflections used in refinement	37 098 (3 671)	41 159 (2 852)	90 055 (8 825)
Reflections used for R-free	1 828 (181)	1 970 (150)	4 406 (437)
R-work	0.16 (0.34)	0.15 (0.21)	0.20 (0.35)
R-free	0.19 (0.40)	0.19 (0.29)	0.25 (0.37)
Number of non-hydrogen atoms	1 241	2 150	7 167
Macromolecules	1 100	1 967	6 424
Solvent	141	167	743
RMS(bonds)	0,008	0,01	0,002
RMS(angles)	0,96	1,12	0,46
Ramachandran favored (%)	99,24	98,27	97,32
Ramachandran allowed (%)	0,76	1,33	2,68
Ramachandran outliers (%)	0	0	0
Average B-factor	26,4	20,3	24,6
Macromolecules	24,9	19,5	23,8
Solvent	37,7	18	31,7

In summary, SCOC LIR domain binding to GABARAP is stabilized not only by core LIR residues but also by multiple interactions involving residues of the N- and C-terminal regions flanking the LIR motif, which range from residues in position X_−8_ to X_+5_. Several GABARAP residues interacting with the SCOC LIR motif are only conserved among GABARAP subfamily proteins (Supplementary Figure 2F).

### ULK kinases and TBK1 phosphorylate SCOC *in vitro*

**Figure 3:**
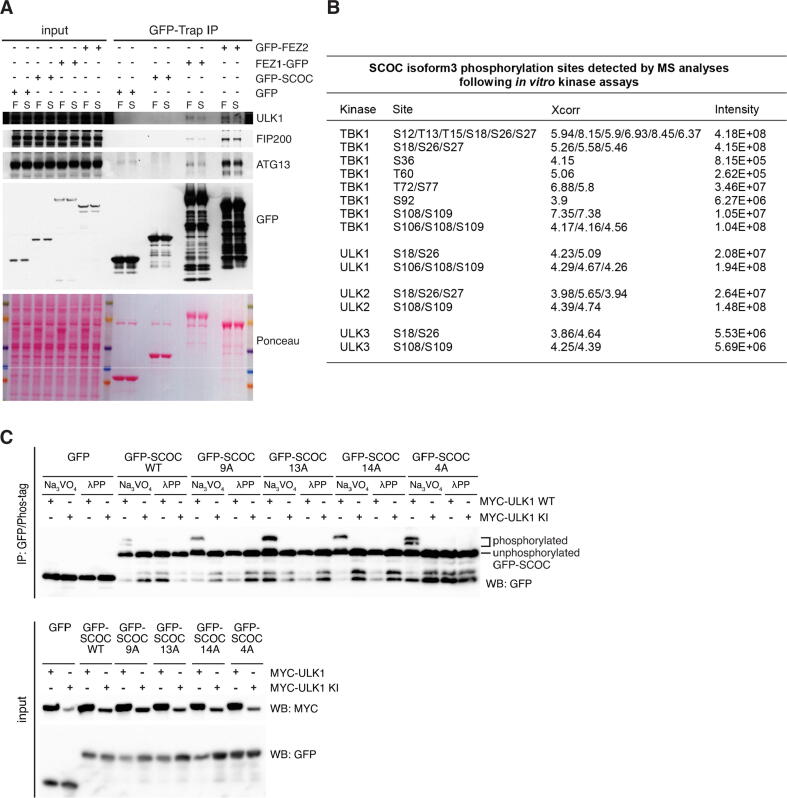
ULK kinases (ULK1-3) and TBK1 phosphorylate SCOC *in vitro*. **A.** GFP-Trap immunoprecipitation (IP) of GFP, GFP-SCOC (isoform 3), FEZ1-GFP or GFP-FEZ2 proteins from HEK293A cells and immunoblots with indicated antibodies. Cells were cultured in full medium (F) or Earle’s balanced salt solution (starvation medium (S)) for 2 h prior to lysis. **B.** Phosphorylation sites detected by mass spectrometry in GST-SCOC (isoform 3) after *in vitro* kinase assays using either recombinant TBK1, ULK1, ULK2 or ULK3. Posttranslational modification mapping was done using the Discoverer 2.4 software (ThermoFisher) and a threshold score (Xcorr) > 2.0. **C.** Western blot analysis of immunoprecipitated GFP, GFP-SCOC (isoform 3) WT, GFP-SCOC (isoform 3) 9A mutant (GFP-SCOC S26A, S27A, S36A, T60A, T72A, S77A, S106A, S108A, S109A), GFP-SCOC (isoform 3) 13A mutant (GFP-SCOC S12A, T13A, T15A, S18A, S26A, S27A, S36A, T60A, T72A, S77A, S106A, S108A, S109A), GFP-SCOC (isoform 3) 14A mutant (GFP-SCOC S12A, T13A, T15A, S18A, S26A, S27A, S36A, T60A, T72A, S77A, S92A, S106A, S108A, S109A) and GFP SCOC (isoform 3) 4A mutant (S12A, T13A, T15A, S18A) using Phos-tag^TM^ SDS-Page. HEK293A cells expressing GFP, GFP-SCOC WT or mutants together with murine MYC-ULK1 wild type (WT) or MYC-ULK1 kinase inactive (KI) (MYC-ULK1-K46I) were starved in Earle’s balanced salt solution for 2 h prior to lysis and GFP-Trap IP. Half of the IP reaction was treated with λ-phosphatase prior to Phos-tag^TM^ SDS-Page and Western blotting. Lower panel shows input analysed by SDS-PAGE and Western blotting.

Phosphorylation of residues in position X_−1,−2_ have been shown to increase LIR binding.^20^, ^32^, ^33^, ^36^ Serine (S12^SCOC^) and threonine (T13^SCOC^) residues precede the SCOC core LIR motif, and moreover, within and directly after the core LIR motif (position X_1_ and X_4_) are serine and threonine residues (T15^SCOC^ and S18^SCOC^), which could be modified by phosphorylation. In Drosophila and mammalian cells, UNC-51 and ULK1, respectively, bind and phosphorylate UNC-76/FEZ1 regulating axonal transport.^39^, ^46^ In line with our previous findings,^39^ GFP-Trap immunoprecipitation experiments showed that FEZ1-GFP but not GFP-SCOC (isoform 3) interacted with the endogenous ULK1 kinase complex members ULK1, ATG13 and FIP200 in both fed and starved HEK293A cells (Figure 3A, and ^39^). Moreover, we detected interaction of GFP-FEZ2 with the ULK1 kinase complex (Figure 3A). FEZ1 and FEZ2 share high sequence conservation between each other, and SCOC (isoform 3) interacted with both FEZ1 (39) and FEZ2 (Supplementary Figure 3A, ^52^).

As we have shown ULK1, FEZ1 and SCOC form a complex (39), we asked if SCOC is phosphorylated by ULK1 through its interaction with FEZ1 and FEZ2. We performed *in-vitro* kinase assays to determine whether recombinant ULK1 can directly phosphorylate SCOC. We also included ULK2 and ULK3 due to potential overlapping functions between these kinases.^6^, ^50^ Furthermore, we tested TBK1, since this kinase has been reported to phosphorylate the LIR domain of OPTN, enhancing OPTN binding to LC3 and clearance of intracellular Salmonella.^32^ Mass spectrometry analyses revealed that *in vitro* experiments all four kinases phosphorylated multiple sites of SCOC isoform 3 (Figure 3B, Supplementary Figure 3B). TBK1 was most active on SCOC isoform 3 with 14 different phosphorylation events. Four to five phosphorylation events were also detected for the three ULK kinases and the identified sites strongly overlapped (S18/S26/S108/S109), indicating similar substrate specificities between ULK1-3. Interestingly, four serine/threonine residues are located within or adjacent to the SCOC core LIR motif (S12/T13/T15/S18^SCOC^). TBK1 again displayed strongest activity on these SCOC LIR motif sites, since we detected phosphorylation of all four serine/threonine residues (S12/T13/T15/S18^SCOC^) as well as the highest intensity values for the corresponding N-terminal peptides. All three ULK kinases phosphorylated S18^SCOC^ in position X_+4_ of the SCOC LIR motif with ULK1 and ULK2 exhibiting higher activities than ULK3.

To test whether ULK1 phosphorylates SCOC in cells, we expressed GFP-tagged SCOC WT together with MYC-tagged ULK1 WT or ULK1 kinase inactive (KI) in HEK293A cells. GFP-tagged proteins were immunoprecipitated using GFP-Trap and half of the IP reaction was subjected to λ-phosphatase treatment (generating non-phosphorylated GFP-SCOC). SCOC phosphorylation was visualised by Western blotting after Phos-tag^TM^ SDS-Page. The Phos-tag reagent reduces electrophoretic mobility of phosphorylated proteins and shifts them to higher molecular weight.^53^ Overexpression of WT but not KI ULK1 induced higher molecular weight shifts of GFP-SCOC (Figure 3C), but no upward shift of GFP. Multiple bands were detected for GFP-SCOC, indicating ULK1 overexpression results in two differentially phosphorylated SCOC species. To determine whether ULK1 phosphorylates residues of the SCOC LIR domain, we mutated all the ULK phosphorylation sites in SCOC identified by mass spectrometry (Figure 3B) to alanine except for those located in the LIR motif and flanking region (S12/T13/T15/S18^SCOC^). Moreover, we also mutated the majority of sites that were identified for TBK1 (Figure 3C). One higher molecular weight band was still detected after expression of the SCOC 9A mutant (S26A, S27A, S36A, T60A, T72A, S77A, S106A, S108A, S109A). However, mutation of the LIR domain residues (referred to SCOC 13A mutant) as well as S92A (referred to SCOC 14A) did not abolish the higher molecular weight band, indicating that ULK1 does not phosphorylate the LIR domain residues and might phosphorylate other sites not detected by mass spectrometry. Alternatively, ULK1 overexpression might lead to activation of other kinases. Equally, mutation of the LIR domain residues S12A, T13A, T15A, S18A alone (referred to SCOC 4A) did not result in loss of the higher molecular weight bands compared to SCOC WT.

Thus, ULK (ULK1-3) kinases and TBK1 phosphorylated SCOC LIR domain residues *in vitro*, but in cells (under overexpression conditions) ULK1 does not phosphorylate the SCOC LIR domain.

### SCOC LIR affinity and specificity is modulated by phosphorylation of residues within and adjacent to the core LIR motif

**Figure 4:**
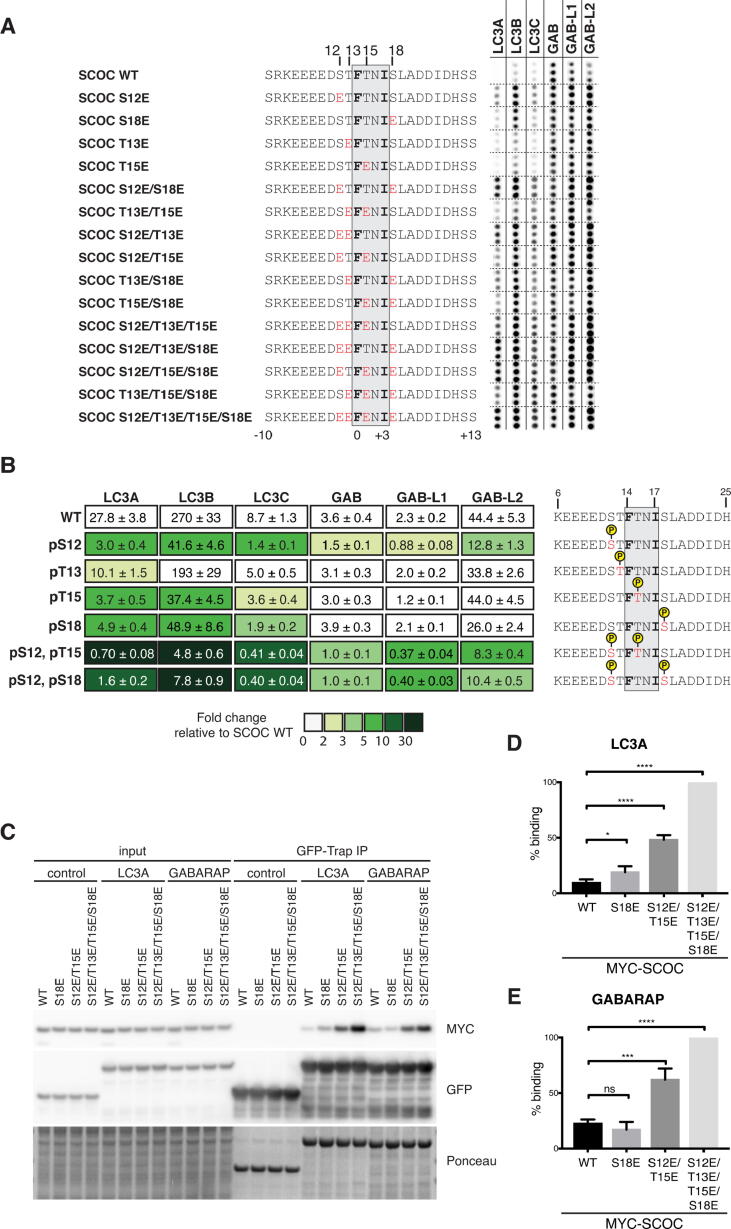
Phosphorylation of the SCOC LIR domain modulates ATG8 binding affinity and specificity. A. Twenty four-mer peptide array of SCOC LIR peptides containing phospho-mimetic mutations incubated with indicated GST-ATG8 protein and immunoblotted with anti-GST. Each peptide is spotted in triplicates. Mutated residues are highlighted in red. B. Affinities (Kd values) of wild-type (WT) and phosphorylated SCOC LIR peptides determined by bio-layer interferometry (BLI). Colour code indicates fold changes relative to Kd value of SCOC WT LIR peptide binding to the corresponding ATG8 protein. Yellow circles indicate phosphorylation modification in SCOC LIR peptide. (data are average Kd values ± standard error calculated from non-linear regression curve fits, n = 2–3). C. GFP-Trap immunoprecipitation (IP) of indicated GFP-ATG8 proteins and immunoprecipitated MYC-tagged SCOC from HEK293A cells analysed by Western blots. To ensure equal amounts of tagged protein are present in each reaction, MYC-tagged SCOC (isoform 3) proteins and GFP-tagged ATG8 proteins were overexpressed separately and lysates were mixed prior to performing the IP experiments. D. and E. Quantification of WT and phospho-mimetic mutant, MYC-tagged SCOC (isoform 3) protein binding to GFP-LC3A (D.) and GFP-GABARAP (E.). Statistical analysis using one-way analysis of variance (ANOVA) test; mean ± s.d.; data from at least three independent experiments (n = 4 [GFP-LC3A]; n = 3 [GFP-GABARAP]); ****p ≤ 0.0001; ***p ≤ 0.001; *p ≤ 0.05.

To determine whether phosphorylation alters binding of ATG8 proteins to the SCOC LIR motif, we performed a peptide array experiment using SCOC LIR domain peptide sequences carrying single as well as multiple phospho-mimetic mutations of S12E^SCOC^, T13E^SCOC^, T15E^SCOC^ and S18E^SCOC^ (Figure 4A). Single point mutations in the SCOC LIR peptide sequence affected mainly the binding of LC3A and LC3B. Mutation of S12^SCOC^ and S18^SCOC^ to glutamate enhanced both GST-LC3B and GST-LC3A binding. The single point mutations T13E^SCOC^ and T15E^SCOC^ also increased GST-LC3A binding to SCOC LIR peptides. The S12E/S18E^SCOC^ or S12E/T13E^SCOC^ double mutation of the SCOC LIR peptide improved both LC3 and GABARAP subfamily binding. The strongest increase in ATG8 binding was detected for the S12E/T13E/S18E^SCOC^, S12E/T15E/S18E^SCOC^ triple mutation as well as mutation of all four residues (S12E/T13E/T15E/S18E^SCOC^), indicating additive effects when combined.

To better understand how phosphorylation of these LIR residues alters the affinity of the SCOC LIR motif to individual ATG8 proteins, we next performed BLI affinity measurements using phosphorylated SCOC LIR peptides (Figure 4B). Consistent with previous findings on the LIR domain of NIX,^33^ phosphorylation of S12^SCOC^ preceding the LIR motif (position X_−2_) strongly increased LC3 subfamily binding by six- to nine-fold. GABARAP subfamily binding was also enhanced two-fold for GABARAP/GABARAPL1 and three-fold for GABARAPL2. However, T13^SCOC^ phosphorylation only slightly improved SCOC LIR binding (two-fold) to LC3A. Phosphorylation of T15^SCOC^ and S18^SCOC^ resulted in seven- to five-fold increase in LC3A and LC3B binding as well as two- to four-fold increase in LC3C binding, whereas no or less than 2-fold changes in affinity were observed for the GABARAPs. Double mutation of S12/T15^SCOC^ and S12/S18^SCOC^ had additive effects on LC3 subfamily binding and particularly, the KD values of LC3A and LC3B changed dramatically (LC3A: 40- and 17-fold, LC3B: 56- and 34-fold, respectively). A 21-fold increase in affinity was observed for LC3C, while the affinity of GABARAP proteins only improved three- to six-fold.

Consistently, phospho-mimetic mutation S18E^SCOC^ increased MYC-SCOC binding to GFP-LC3A in GFP-Trap IP experiments (Figure 4C, 4D), but did not alter MYC-SCOC interaction with GFP-GABARAP (Figure 4C, 4E). MYC-SCOC S12E/S18E bound significantly more to both GFP-LC3A and GFP-GABARAP and the strongest binding was detected when phospho-mimetic mutations were introduced for all four potential phosphorylation sites (Figure 4C-E), further confirming additive effects of these sites.

In summary, analysis of potential phosphorylation sites in the SCOC LIR domain in position X_−1_, X_−2_, X_1_ and X_4_ revealed that not only phosphorylation of position X_−2_ preceding the core LIR motif, but also positions X_1_ and X_4_ (located within and C-terminal to the core LIR motif) selectively enhance LC3 subfamily binding and that these residues are critical in regulating binding specificity of the SCOC LIR motif. Interestingly, phosphorylation of S12/T15^SCOC^ and S12/S18^SCOC^ had a substantial effect on LC3 binding promoting it from very weak to strong binding suggestive of an important regulatory function in vivo.

### Structure of the phosphorylated SCOC LIR domain in complex with GABARAPL1

**Figure 5:**
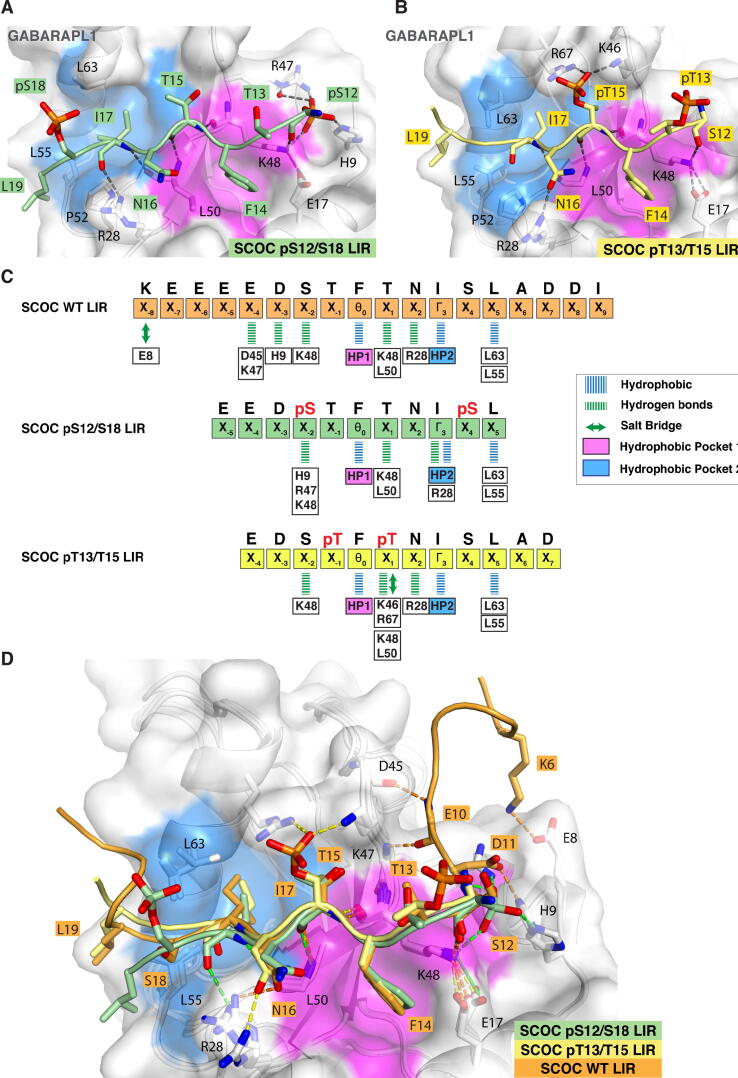
Structure of the phosphorylated SCOC LIR domain in complex with GABARAPL1. A. Structure of the phosphorylated SCOC pS12/S18 LIR domain peptide (aa 9–19) bound to GABARAPL1. The SCOC LIR peptide sequence is shown in light green cartoon with interacting residues depicted as sticks. GABARAPL1 is displayed in white cartoon and transparent surface with hydrophobic pocket 1 (pink) and 2 (blue). B. Structure of the phosphorylated SCOC pT13/T15 LIR domain peptide (aa 10–21) bound to GABARAPL1. The SCOC LIR sequence is shown in yellow cartoon with interacting residues depicted as sticks. C. Schematic overview of LIR interactions observed in the structures of GABARAP bound to SCOC WT LIR domain (orange), and GABARAPL1 bound to SCOC pS12/S18 (light green) and SCOC pT13/T15 (yellow) LIR domains. GABARAP/GABARAPL1 residues are boxed and shown in black. Blue lines indicate hydrophobic interactions, green lines hydrogen bonds, and green double arrow salt bridges. Pink and blue boxes indicate canonical LIR interactions (hydrophobic contacts) with HP1 and HP2. D. Superposition of the structures of GABARAP bound to SCOC WT (orange), and GABARAPL1 bound to SCOC pS12/S18 (light green) and SCOC pT13/T15 (yellow) LIR domains. Only the surface of GABARAP from the GABARAP:SCOC WT LIR complex structure is displayed in white transparency with HP1 and HP2 coloured in pink and blue, respectively.

Next, we tried to co-crystalise GABARAPL1, LC3C and LC3A with phosphorylated SCOC LIR peptides to solve the structures of phosphorylated SCOC LIR:ATG8 complexes. GABARAPL1 was used instead of GABARAP, since purified GABARAP aggregated during concentration. Our attempts to co-crystalise SCOC-2pS and SCOC-2pT peptides in complex with LC3A and LC3C, were also unsuccessful, but we were able to determine the crystal structure of a SCOC LIR domain peptide phosphorylated at S12^SCOC^ and S18^SCOC^ (referred to as SCOC-2pS hereafter) in complex with GABARAPL1 at a resolution of 1.45 Å (Figure 5A, Supplementary Figure 3C) as well as the structure of a SCOC LIR domain peptide phosphorylated at T13^SCOC^ and T15^SCOC^ (referred to as SCOC-2pT) in complex with GABARAPL1 at a resolution of 1.72 Å (Figure 5B, Supplementary Figure 3D).

In both structures SCOC formed the same canonical LIR interactions with GABARAPL1 as described previously for the SCOC WT LIR-GABARAP complex (Figure 2C). Furthermore, we observed multiple additional contacts in both structures: Hydrogen bonds were formed between the carbonyl of pS12^SCOC-2pS^/S12^SCOC-2pT^ and K48^GABL1^, as well as between R28^GABL1^ and the carbonyl of I17^SCOC-2pS^ or the sidechain of N16^SCOC-2pT^ (Figure 5 A-C). Moreover, we detected some hydrophobic contacts between L19^SCOC-2pS/2pT^ and the edge of HP2. In the SCOC-2pS LIR, the phosphoserine pS12 formed a large network of electrostatic interactions with H9^GABL1^, K48^GABL1^ and R47^GABL1^, while pS18 was not interacting with GABARAPL1. Similarly, in the SCOC-2pT LIR crystal structure, pT13 was not interacting with GABARAPL1, but pT15 in position X_1_ formed salt bridges with K46^GABL1^ and R67^GABL1^.

The leucine residue L19^SCOC^ of SCOC (position X_+5_) also formed hydrophobic interactions with L55^GAB/GABL1^ and L63^GAB/GABL1^ of GABARAP in the SCOC WT-LIR:GABARAP complex (Figure 2C,D and Figure 5C). In mutational peptide array scans (Figure 2A, B and Supplementary Figure 2D) only substitutions of L19^SCOC^ with aromatic aa (W, F, Y) or hydrophobic aa (I) resulted in substantial ATG8 binding, indicating that hydrophobic contacts mediated by the LIR residue in position X_+5_ were important for both GABARAP and LC3 subfamily binding.

Whereas more distant N-terminal residues (K6^SCOC^ (X_−8_), E10^SCOC^ (X_−4_) and D11^SCOC^ (X_−3_)) contributed to binding of the SCOC WT LIR motif to GABARAP, no involvement of E10^SCOC^ (X_−4_) and D11^SCOC^ (X_−3_) (which were present in the phosphorylated peptides) was detected in the structures of the SCOC-2pS:GABARAPL1 and SCOC-2pT:GABARAPL1 complexes (Figure 5 C-D).

### Distant flanking residues in the SCOC LIR motif contribute to ATG8 binding

**Figure 6:**
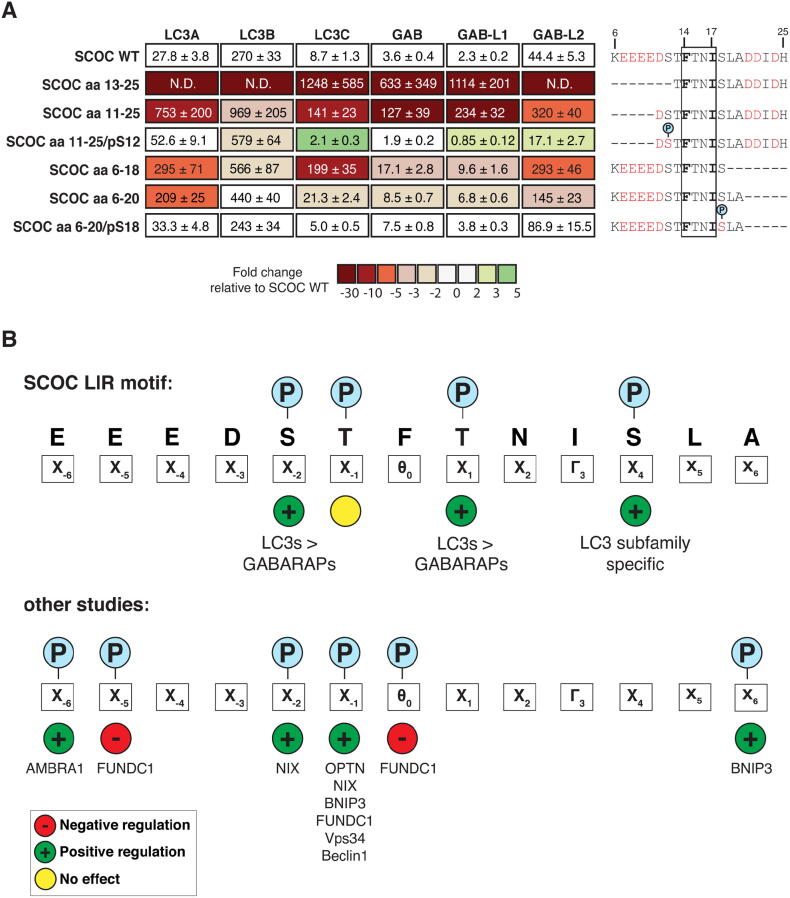
Contribution of the flanking regions in the SCOC LIR domain to ATG8 binding. **A.** Affinities (Kd values) of wild-type (WT), truncated and phosphorylated SCOC LIR domain peptides determined by bio-layer interferometry (BLI). Colour code indicates fold changes relative to Kd value of SCOC WT LIR peptide binding to the corresponding ATG8 protein. Blue circles indicate phosphorylation site in SCOC LIR sequence. Acidic residues are highlighted in red. (data are average Kd values ± standard error calculated from non-linear regression curve fits, n = 2). **B.** Schematic overview of regulatory phosphorylation sites identified in SCOC and other LIR motifs. Red circle with minus sign indicates negative regulation, green circle with plus sign positive regulation and yellow circle no regulation of ATG8 binding.

To further refine the data and elucidate the importance of the flanking residues in regulating SCOC LIR binding, we performed BLI affinity measurements (Figure 6A). Removal of the N-terminal sequence (except for T13^SCOC^) resulted in a profound decrease (more than 140-fold) in ATG8 binding to the SCOC LIR motif (aa 13–25). Loss of the N-terminal lysine and glutamate residues in the SCOC LIR motif (aa 11–25) also strongly reduced interaction with LC3A (27-fold), LC3C (16-fold), GABARAP (37-fold) and GABARAPL1 (104-fold). Equivalently, truncation of the C-terminal flanking residues of the SCOC LIR motif (except for S18^SCOC^) resulted in a substantial decrease in affinity ranging from four-fold for GABARAP and GABARAPL1 to 10-fold and 22-fold for LC3A and LC3C, respectively. Loss of the C-terminal aspartate, isoleucine and histidine residues in the SCOC LIR motif (aa 6–20) lead to a two- to three-fold reduction binding to GABARAPs and LC3C and a more than seven-fold reduction in LC3A binding. Interestingly, both phosphorylation of S12^SCOC^ and S18^SCOC^ rescued ATG8 binding of the SCOC LIR motif, indicating that phosphorylation of these residues can compensate the loss of flanking residues and positively regulates SCOC LIR binding to ATG8 proteins.

## Discussion

Phosphorylation of LIR motifs of autophagy receptors and adaptors plays an important role in regulating selective autophagy pathways, e.g. mitophagy^33^, ^34^, ^35^, ^36^, ^37^, ^38^ and xenophagy.^32^ Our understanding of the underlying regulatory mechanisms, i.e. kinases mediating LIR motif phosphorylation as well as key phosphorylation sites in LIR motifs modulating ATG8 binding affinity and specificity, is still limited. Several studies have shown that phosphorylation of residues directly preceding the core LIR motif (in position X_−1_, X_−2_, and X_−6_) enhance ATG8 binding^20^, ^32^, ^33^, ^34^, ^36^, ^38^ (Figure 6B). Inhibition of LC3 binding has been shown for phosphorylation of the aromatic core LIR residue Y18^FUNDC1^ (Θ_0_)^35^ and the N-terminal residue S13^FUNDC1^ (X_−5_)^37^ of the mitophagy receptor FUNDC1. Whether LIR-ATG8 interactions are regulated by phosphorylation of other residues within or C-terminal to the core LIR motif still remains elusive.

In this study we identified a functional LIR motif in the small Golgi protein SCOC (isoform 3), which was phosphorylated by ULK kinases (ULK1-3) as well as TBK1 at serine/threonine residues immediately flanking or within the core LIR motif of SCOC (S12/T13/T15/S18^SCOC^). To determine and understand the regulatory effects of these phosphorylation sites on SCOC LIR binding affinity and specificity towards all six mammalian ATG8 proteins we carried out a comprehensive analysis involving high resolution crystal structures, peptide array and IP binding assays, as well as BLI affinity measurements.

Phosphorylation of S12^SCOC^ in position X_−2_ of the SCOC LIR motif increased ATG8 binding (Figure 4A, B). Surprisingly, phosphorylation of T13^SCOC^ had little or no effect on ATG8 binding affinities although phosphorylation of X-_1_ residues or substitution with D/E (mimicking phosphorylation) has been widely reported to positively regulate interaction with ATG8 proteins^20^, ^32^, ^33^, ^34^, ^35^, ^36^ (Figure 6B). In our crystal structure phosphorylated S12^SCOC^ formed a large network of hydrogen bonds with H9^GABL1^, K48^GABL1^ and R47^GABL1^, whereas phosphorylated T13^SCOC^ was solvent exposed and not interacting with LDS amino acids of GABARAPL1 (Figure 5A-C). This lack of interaction explains why phosphorylation of T13^SCOC^ of the SCOC LIR domain did not enhance GABARAP subfamily binding (Figure 4B).

S12^SCOC^ phosphorylation enhanced the affinity of the SCOC LIR motif to LC3s rather than GABARAPs (Figure 4B). Since the LDS of LC3 subfamily proteins has more basic residues at the edge of HP1 (R10/R11^LC3A/B^ and R16/K17^LC3C^ on helix α1), a larger network of electrostatic interactions with acidic or phosphorylated LIR residues in position X_−2_ to X_−4_ can be formed,^27^, ^30^, ^54^, ^55^, ^56^, ^57^ which promotes more binding to LC3s than GABARAPs.

Phosphorylation of T15^SCOC^ at position X_1_ of the SCOC LIR motif also increased much stronger the binding affinities of LC3 subfamily proteins (especially LC3A and LC3B) than of GABARAPs (Figure 4B). In the WT SCOC LIR:GABARAP complex structure two hydrogen bonds were formed between the main chain residues of T15^SCOC^ and K48^GAB^ and L50^GAB^ (Figure 2C, D). In the SCOC-2pT LIR:GABARAPL1 complex structure phosphorylated T15^SCOC^ formed two additional salt bridges with K46^GABL1^ and R67^GABL1^ of GABARAPL1 (Figure 5B). Both K46^GABL1^ and R67^GABL1^ are conserved in both LC3 and GABARAP proteins (Supplementary Figure 2F). Additional electrostatic interactions therefore may also occur between phosphorylated T15^SCOC^ and the corresponding residues (K49^LC3A/B^/K55^LC3C^ and R70^LC3A/B^/R76^LC3C^) in LC3 subfamily proteins. Interestingly, in a compilation of 100 LIR sequences E is the residue most frequently found in position X_1_ followed by V and D.^5^ Several LIR-ATG8 complex structures of LIR motifs with E/D in position X_1_ have been solved. Salt bridges between E in position X_1_ and R67^GAB/GABL1^ are observed in the structures of the ATG4B LIR:GABARAPL1,^22^ VPS34 LIR:GABARAP, and ATG14-LIR:GABARAP^20^ complexes. Electrostatic interactions between E/D in position X_1_ and R70^LC3B^ (corresponding to R67^GAB^ of GABARAP) are also reported for the TECPR2 LIR:LC3B,^58^ FYCO1 LIR:LC3B,^54^, ^55^, ^56^ and RavZ LIR2:LC3B^59^ complex structures. Thus, acidic or phosphorylated residues in position X_1_ seem to generally stabilize LIR-ATG8 interactions. We currently do not understand why these additional electrostatic interactions significantly increase the affinity of the pT15 SCOC LIR motif to LC3s more than GABARAPs (Figure 4B) and this needs further investigation.

Growing evidence indicates that the region C-terminal to the core LIR motif is involved in ATG8 binding and can even regulate ATG8 binding specificity.^30^, ^54^, ^55^, ^56^, ^60^, ^61^ We recently demonstrated a critical role of the X_4_ LIR domain residue in regulating ATG8 binding specificity.^30^ The proline residue in position X_4_ specifically impairs binding of the ULK1 and FIP200 LIR motifs to LC3s, whereas mutation of this residue to D (P361D^ULK1^ and P706D^FIP200^) improves it. The LIR domain of the centrosomal satellite protein PCM1 has an E (E1959^PCM1^) in position X_4_. Mutation of E1959^PCM1^ to T specifically decreases the affinity of the PCM1 LIR motif to LC3s, but does not alter the affinity to GABARAP proteins.^30^ In support of a role for X_4_ in generating ATG8 specificity, phosphorylation of S18^SCOC^ in position X_4_ of the SCOC LIR domain also increased LC3 subfamily binding (Figure 4B, C). Notably, in the SCOC-2pS LIR:GABARAPL1 complex structure phosphorylated S18^SCOC^ was solvent exposed and not interacting with GABARAPL1 (Figure 5A). Similarly, phosphorylation of the LIR motif of the cysteine protease ATG4B at position X_4_ (S392^ATG4B^), potentially implicated in LC3 delipidation^50^, does not contribute to GABARAPL1 binding.^22^ However, in the PCM1 LIR:GABARAP complex structure E1959^PCM1^ forms a salt bridge with R28^GAB^ of GABARAP.^30^ Both GABARAP and LC3 subfamily proteins have a basic residue (R28^GAB/GABL1/GABL2^, K30^LC3A/LC3B^ or K36^LC3C^) in the LDS that could potentially engage with the phosphorylated LIR residue in position X_4_. To further understand the binding mechanism of this region of the LIR domain, more structural data of C-terminally extended LIR motifs (with E/D/pS/T in position X_4_) in complex with LC3 proteins are needed.

Whereas electrostatic interactions between GABARAP and acidic residues in the N-terminal region of the SCOC LIR domain were observed, we did not detect interactions of acidic residues C-terminal (position X_7_ to X_10_) to the core LIR motif. Removal of C-terminal residues more distant than L19 (SCOC aa 20–27) reduced binding (Figure 6A), suggesting that these residues are important for ATG8 binding. However, electrostatic side chain and/or backbone interactions of these residues with the LDS seem to be highly dynamic and difficult to detect by structural analyses such as X-ray crystallography.

Notably, phosphorylation of S18^SCOC^ rescued binding to GABARAPs and LC3s in the absence of the C-terminal SCOC LIR motif residues in position X_7_ to X_11_ (aa 21–25) (Figure 6A), suggesting that pS18^SCOC^ can contribute or stabilize GABARAP and LC3 binding if interactions with more C-terminally located LIR motif residues are weak or weakened. Similarly, S12^SCOC^ phosphorylation rescued ATG8 binding after deletion of aa 6–10 (positon X_−4_ to X_−8_) (Figure 6A). In the SCOC-2pS LIR:GABARAPL1 structure electrostatic interactions with the more distant SCOC LIR domain residues (E10^SCOC^ (X_−4_) and D11^SCOC^ (X_−3_)) were not detected and may not be required for LIR binding to GABARAPL1 due to additional interactions established by phosphorylated S12^SCOC^.

In summary, our data demonstrates that there is high plasticity and dynamics in the interactions that the LDS of ATG8 proteins forms with the extended N- and C-terminal regions flanking the core LIR motif. We identified novel regulatory phosphorylation sites within and C-terminal to the core LIR motif of SCOC. ATG8 binding affinity and specificity of other LIR motifs could potentially also be positively regulated by phosphorylation of these positions.

Lastly, autophagy-independent functions have been reported for ULK kinases,^46^, ^62^ GABARAP,^63^ SCOC and FEZ1.^41^, ^44^, ^45^, ^64^ SCOC is not degraded by autophagy (Supplementary Figure 2B and C) and therefore possibly functions as an autophagy adaptor protein and/or in autophagy-independent processes. Through its interaction with the kinesin1 adaptor protein FEZ1^39^, ^41^, ^42^, ^43^ SCOC might be involved in microtubule-dependent transport of ATG8 proteins and autophagosomal membranes in neurons.

Phosphorylation of both S12^SCOC^ and T15^SCOC^ or S18^SCOC^ resulted in a 34 to 56-fold increase in LC3B binding affinity with potential relevance in cells (Figure 4B). As the phos-tag gel assay to demonstrate ULK1 phosphorylation of the SCOC LIR domain in HEK293A cells (Figure 3C) did not confirm the in vitro kinase assay results (Figure 3B), further analyses are needed to determine whether this phosphoregulation of LC3B binding occurs in cells. Understanding the role of SCOC in neuronal autophagy and axonal transport will be an interesting direction for future research.

## Methods

### Antibodies

The following primary antibodies were used: HRP-conjugated anti-GST (GE Healthcare, RPN1236); anti-MYC (CRUK, 9E10; Abcam, ab9106); anti-GFP (CRUK, 3E1; Santa Cruz, sc-8334); anti-GABARAP (Abgent, AP1821a); anti-actin (Abcam, ab8227), anti-FLAG M2 (SIGMA, F1804), anti-TGN46 (Serotec, AHP500), anti-beta-tubulin (Abcam, ab6046), anti-ULK1 (Santa Cruz, sc-33182); anti-ATG13^65^; anti-FIP200 (Bethyl Labs, A301-536A-1); anti-FEZ2 (SIGMA, HPA0355978). Secondary antibodies for IF were: anti-rabbit IgG Alexa Fluor 555 and 647; anti-mouse IgG Alexa Fluor 555, 647; anti-sheep IgG Alexa Fluor 647 (all from Life Technologies). HRP-conjugated secondary antibodies used for WB were from GE Healthcare.

### Plasmids

pDEST-EGFP, pDEST-EGFP-LC3A, pDEST-EGFP-GABARAP, pDEST15-ATG8 homologs (GST-tagged human ATG8 homologs), pENTRY-SCOC (isoform 3), pDEST-FLAG-SCOC (isoform 3) and pDEST-MYC-SCOC (isoform 3) were generated by the laboratory of Terje Johansen (UiT, The Arctic University of Norway, Tromsø).^17^, ^18^, ^39^, ^66^ pENTRY-SCOC F14A/I17A (isoform 3), pENTRY SCOC S18E, S12E/S18, and S12E/T13E/T15E/S18E (isoform 3) were generated using the QuickChange Site-Directed Mutagenesis Kit (Stratagene). Gateway destination plasmids were made using Gateway LR recombination reactions (Invitrogen) and the pDEST-MYC vector (N-terminal MYC-tag) according to manufacturer’s instructions. STOP codons were introduced at the end of the SCOC (isoform 3) sequences using the QuickChange Multi Site-Directed Mutagenesis kit (Agilent). FEZ2 cDNA was amplified from a HeLa cDNA library and cloned into the pENTRY1A vector. pDEST-EGFP-FEZ2 was obtained from the pENTRY1A-FEZ2 by GATEWAY LR reaction.

Human ATG8 homologs in the pAL plasmid (containing N-terminal GST-tag followed by a 3C protease cleavage site)^30^ were used to recombinantly express ATG8 proteins for BLI affinity measurements. For crystallization, the SCOC wild type LIR sequence (aa 6–23) were N-terminally fused with a Gly-Ser linker to full-length GABARAP (GST-GABARAP (pAL)) using NcoI and BamHI sites. The QuickChange Multi Site-Directed Mutagenesis Kit (Agilent) was used to introduce the S113Stop^GABARAP^ mutation and remove the last five aa from the C-terminus of GABARAP.

The pEGFPN2-FEZ1 expression plasmid^67^ was kindly provided by Caroline Whitehouse (King’s College, UK). N-terminal MYC-tagged wild type and kinase-dead (K46I) murine ULK1 expression plasmids (pRK5)^68^ as well as pEGFPC1-SCOC^39^ expression plasmids have been described previously. pEGFPC1-SCOC 9A mutant (SCOC isoform 3 S26A, S27A, S36A, T60A, T72A, S77A, S106A, S108A, S109A), pEGFPC1-SCOC 13A mutant (SCOC isoform 3 S12A, T13A, T15A, S18A, S26A, S27A, S36A, T60A, T72A, S77A, S106A, S108A, S109A), pEGFPC1-SCOC 14A mutant (SCOC isoform 3 S12A, T13A, T15A, S18A, S26A, S27A, S36A, T60A, T72A, S77A, S92A, S106A, S108A, S109A) and pEGFPC1-SCOC 4A mutant (SCOC isoform 3 S12A, T13A, T15A, S18A) expression plasmid were generated using QuickChange Multi Site-Directed Mutagenesis Kit (Agilent) and/or Q5 Site-Directed Mutagenesis Kit (New England BioLabs).

All plasmid constructs generated in this study were verified by DNA sequencing.

### Cell culture and generation of stable cell lines

HEK293A cells (provided by Cell Services of the Francis Crick Institute) were grown in a humidified incubator at 37 °C in 10% CO2 in full medium (Dulbecco’s modified Eagle’s medium supplemented with 10% fetal calf serum and 4 mM l-glutamine). Lipofectamine 2000 (Life Technologies) was used for transient transfection of cells according to the manufacturer’s instructions. DNA plasmids were used at a concentration of 1 mg/ml of transfection mix. Cells were harvested after 24 h.

HeLa Flp-In T-Rex cell lines expressing GFP-SCOC (isoform 1), GFP-SCOC (isoform 3), GFP-SCOC F14A/I17A (isoform 3) from a tetracycline inducible CMV promoter were made using the FlpIn T-Rex system (Thermofisher, R71407). pDest-EGFP-Flp-In SCOC iso1, pDest-EGFP-Flp-In SCOC iso3 and pDest-EGFP-Flp-In SCOC iso3 LIRm constructs were established by GATEWAY cloning into the pDEST-EGFP-FlpIn vector.^18^ The HeLa-based Flp-In T-Rex host cell line (Invitrogen) contains a single integrated FRT site for insertion of selected constructs. To generate stable cell lines, Flp-In plasmids carrying the selected GFP-tagged constructs were co-transfected with pOG44 encoding the Flp-In recombinase in the ratio of 1:3. Cells were selected by treatment with 200 µg/ml Hygromycin B (Invitrogen, #10687010) and 7,5 µg/ml Blasticidine (Gibco, A1113903). Expression from the CMV-TetO2 promoter was induced by adding 0.5 μg/ml of tetracycline (Sigma, T7660) for 16 to 24 hours. HeLa Flp-In T-Rex cell lines were maintained in Dulbecco’s modified Eagle’s medium supplemented with 10% fetal calf serum, 4 mM l-glutamine, 1% streptomycin-penicillin, 100 µg/ml Hygromycin B, and 3.5 µg/ml Blasticitidine. Cells were treated as indicated with 100 nM Bafilomycin A1, 10 µM MG132, or 1 µM epoxomicin in full medium or Earle’s balanced salt solution (EBSS).

### Western blotting

Cells were lysed in ice-cold TNTE buffer (20 mM Tris-HCl, pH 7.4, 150 mM NaCl, 0.5% w/v Triton X-100, 10% v/v glycerol, 5 mM EDTA) containing EDTA-free Complete Protease Inhibitor cocktail (Roche) and PhosSTOP (Roche). Lysates were cleared by centrifugation and resolved on NuPAGE Bis-Tris 4–12% gels (Life Technologies) followed by transfer onto a PVDF membrane (Millipore). After incubation with primary and secondary antibodies, the blots were developed by chemiluminescence using Immobilon Classico Western HRP substrate (Merck Millipore) or enhanced chemiluminescence reagents (GE Healthcare). Densitometry was performed with ImageJ software. For western blotting of weak signal antibodies, primary antibody was diluted with SignalBoost Immunoreaction Enhancer Kit (Merck Millipore, 407207) and blots were developed with Luminata Crescendo Western HRP substrate (Merck Millipore).

### GST pulldown assay

GST- and GST-tagged proteins were expressed in E. coli LE392 and BL21(DE3), respectively, purified and immobilized on Glutathione Sepharose 4 Fast Flow beads (GE Healthcare). One μg of the appropriate DNA constructs were used to produce full reactions (50 μl) of ^35^S-labeled proteins following the TNT T7 Quick Coupled Transcription/Translation system (Promega). For GST pulldown assays 10 μl of each *in vitro* translated protein diluted in 200 μl of NET-N buffer (20 mM Tris-HCl, pH 8.0, 100 mM NaCl, 1 mM EDTA, 0.5% Nonidet P-40 (v/) containing cOmplete Mini EDTA-free protease inhibitor cocktail (Roche Applied Science)). The binding assay (GST pulldown), gel electrophoresis and visualization of binding by autoradiography was performed as described by Johansen et al. ^69^.

### Immunoprecipitation

For GFP-Trap IP experiments (Figure 4C), cells were lysed in ice-cold TNTE buffer (20 mM Tris-HCl pH 7.4, 150 mM NaCl, 5 mM EDTA, 0.5% w/v Triton X-100, 10% v/v glycerol, 1x Complete protease inhibitor (Roche), 1x PhosSTOP (Roche)). Lysates were cleared by centrifugation at 16,000xg, 4 °C for 15 min. To ensure that equal amounts of overexpressed GFP- and MYC-tagged proteins were present in each reaction, equal volumes of GFP-tagged protein extracts were mixed with equal volumes of MYC-tagged protein extracts. Lysates were precleared with control agarose beads (ChromoTek) for 1 hour at 4 °C. GFP-tagged proteins were immunoprecipitated using GFP-TRAP beads (ChromoTek) overnight at 4 °C. Beads were washed 3 times with TNTE (w/o PhosSTOP) and bound protein was eluted with 2x Laemmli buffer at 100 °C for 10 min before resolving by SDS-PAGE (4%–12% Bis-Tris NuPAGE gels, Life Technologies) and western blotting. GFP-Trap IP (Figure 3A) and FLAG IP (Supplementary Figure 3A) experiments were performed with slight modifications. Cells were lysed in TNTE buffer A (50 mM Tris-HCl pH 7.4, 150 mM NaCl, 5 mM EDTA, 1% w/v Triton X-100, 10% v/v glycerol, 1x Complete protease inhibitor (Roche), 1x PhosSTOP (Roche)), cell lysates were not precleared with control agarose beads and IP reactions were washed with TNTE buffer B (50 mM Tris-HCl pH 7.4, 150 mM NaCl, 5 mM EDTA, 0.1% w/v Triton X-100, 10% v/v glycerol, 1x Complete protease inhibitor (Roche), 1x PhosSTOP (Roche)). FLAG-tagged SCOC was immunocaptured using Anti-FLAG-M2 affinity agarose gel (SIGMA, A2220).

### *In vitro* kinase assay and mass spectrometry

For *in vitro* kinase assay, 50 ng of recombinant kinases [ULK1 (SignalChem, #U01-11G), ULK2 (Merck Millipore, #14-772); ULK3 (Merck Millipore, #14-755); TBK1 (Merck Millipore, #14-628)], 1 µg of GST tagged SCOC isoform 3 and 60 mM ATP and combined in 30 μl of kinase buffer (35.5 mM Tris pH7.5, 10 mM MgCl2, 0.5 mM EGTA pH 8.0, 0.1 mM CaCl2) supplemented with cOmplete Mini EDTA-free protease inhibitor cocktail tablets (Roche Applied Science, #11836170001) and phosphatase inhibitor cocktail (Merck Millipore, #524625) and incubated for 20 minutes at 30 °C. The reaction was stopped by addition of 6xSDS-loading buffer and samples separated by SDS-PAGE. The gels were stained and SCOC band cut out for protease digestion and mass spectrometric analysis. In-gel chymotrypsin digestion was performed before analysis by high-performance liquid chromatography–tandem mass spectrometry (HPLC-MS/MS). Gel pieces were subjected to in-gel reduction, alkylation, and digestion using 6 ng/μl chymotrypsin (V1062; Promega) or Trypsin (V5111; Promega). OMIX C18 tips (Varian) were used for sample cleanup and concentration. Peptide mixtures containing 0.1% formic acid were loaded onto a ThermoFisher Scientific EASY-nLC1200 system. Samples were injected to a trap column (Acclaim PepMap 75 μm × 2 cm, C18, 3 μm, 100 Å; ThermoFisher) for desalting before elution to the separation column (EASY-Spray column, C18, 2 μm, 100 Å, 50 μm, 50 cm; ThermoFisher). Peptides were fractionated using a 4–40% gradient of increasing amounts of 80% Acetonitrile in water over 60 min at a flow rate of 300 nl/min. The mobile phases contained 0.1% formic acid. Separated peptides were analyzed using an Orbitrap Fusion Lumos mass spectrometer. The mass spectrometer was operated in a data-dependent mode with the precursor scan in the orbitrap over the range m/z 350–1500. The most intense ions were selected for ETD or CID fragmentation using 3 sec between each master scan. Dynamic exclusion was set to 8 s. The Orbitrap AGC target was set to 4E5 with maximum injection time 50 ms. The MS2 scans in the Ion Trap was set to 1E4 with dynamic injection time. Precursor ions with charge 3+ in the m/z range 350–650 and 4+ or 5+ ions in the m/z range 350–900 was fragmented with ETD. All ions with 6+ or higher were also fragmented using ETD. The rest of the precursor ions were fragmented using CID. Protein identification and PTM mapping was done using the Proteome Discoverer 2.4 software (ThermoFisher) using the ptmRS module (>75%). Peak lists generated in Proteome Discoverer was searched against the UniProt *Homo sapiens* proteome (april 2019; 73,645 sequences) using the built in Sequest HT search engine. Search parameters were:

**Table:** 

- Enzyme:	Chymotrypsin (Full);
- Max missed cleavage:	2
- Precursor mass tolerance:	10 ppm
- Fragment mass tolerance:	0.6 Da
- Fixed modifications:	Carbamidomethyl (C)
- Dynamic modifications:	Oxidation (M), Phospho (ST), Acetyl (protein N-term), Met-loss (protein N-term), Met-loss + Acetyl (protein N-term)
- Threshold score	Xcorr > 2.0
- #peptides	>2

### Lambda-phosphatase treatment and Phos-tag^TM^-SDS Page

To determine SCOC phosphorylation in vivo (Fig. 3C), myc-ULK1 WT or myc-ULK1 KI were co-expressed along with pEGFPC1, pEGFPC1-SCOC or pEGFPC1-SCOC 9A in HEK293A cells. 24 h after transfection, cells were starved for 2 h with EBSS. Cells were then harvested and washed with cold PBS, and lysed in TNTE buffer (w/o 10% v/v glycerol). The cell lysates were immunoprecipitated using GFP-TRAP (ChromoTek) as decribed above. Prior to lambda-phosphatase treatment, GFP-TRAP beads were washed 3 times with TNTE buffer (w/o 10% v/v glycerol, EDTA and PhosSTOP) and split into two equal portions for λ-phosphatase treatment and control. Lambda phosphatase was added into each sample with phosphatase reaction buffer and MnCl_2_ following manufacturer’s instructions (New England BioLabs). Sodium orthovanadate (Na_3_VO_4_) was added into each sample at the final concentration of 100 μM, as a control. All reactions were incubated at 30 °C for 30 min. 2x Laemmli buffer was added into all the samples to stop reaction and then boiled at 100 °C for 5 min. Samples were separated by 10% SDS-PAGE with 25 μM Phos binding reagent (Phosbind) acrylamide (APExBIO, F4002) and analysed by immunoblotting.

### Peptide array and GST overlay assay

GST or GST-ATG8 proteins were expressed (from GST-ATG8 (pAL) plasmids) in *E. coli* BL21 (DE3) plysS cells (Agilent) in LB medium supplemented with 50 µg/ml kanamycin. Expression was induced by addition of 0.5 mM IPTG at OD_600_ = 0.6 and cells were incubated at 25 °C overnight or at 37 °C for 5 h. Harvested cells were lysed in 50 mM Tris-HCl, pH 8.0, 500 mM NaCl, 0.1% Triton X-100, 0.4 mM AEBSF and 15 µg/ml Benzamidine. Fusion protein was batch-adsorbed onto Glutathione-Sepharose 4B beads (GE Healthcare). After five washes with wash buffer (50 mM Tris, pH 8.0, 250 mM NaCl, 0.4 mM AEBSF and 15 µg/ml benzamidine) fusion proteins were eluted in 50 mM Tris pH 8.0, 2 mM L-glutathione reduced, 0.4 mM AEBSF and 15 µg/ml benzamidine.

A MultiPep or ResPep SL automated synthesizer (INTAVIS Bioanalytical Instruments AG, Germany) was used for SPOT synthesis of peptide arrays on cellulose membranes.^70^ After blocking membranes in TBST with 5% nonfat dry milk, peptide interactions with GST or GST fusion proteins were tested by overlaying the membranes with either 1 µg/ml (mutational peptide array scan) or 2 µg/ml of recombinant protein (all other peptide arrays) for 2 h at room temperature. Membranes were washed in TBST, and bound proteins were detected with HRP-conjugated anti-GST antibody (1:5000, GE Healthcare, RPN1236).

### Protein expression and purification for crystallization

GST-GABARAPL1 full-length (pAL)^30^ or GST-SCOC^6-23^LIR-GABARAP(S113Stop) (pAL) were expressed in *E. coli* Rosetta (DE3) pLysS at 25 °C overnight. Bacteria were harvested by centrifugation and resuspended in lysis buffer (50 mM Tris-HCl, pH 8.0, 500 mM NaCl, 0.1% TX-100, 0.5 mM TCEP, 0.5 mM AEBSF and 15 ug/ml benzamidine). The fusion proteins were batch-adsorbed onto a glutathione-Sepharose affinity matrix and recovered by cleavage with 3C protease at 4 °C overnight in 50 mM Tris-HCl, pH 8.0, 100 mM NaCl, 0.5 mM TCEP. The protein was then purified by size exclusion chromatography using a Superdex 75 column equilibrated and run in 25 mM Tris-HCl, pH 8.0, 150 mM NaCl and 0.5 mM TCEP. Peptides were synthesized by the Francis CRICK Institute peptide chemistry science technology platform.

### Crystallisation and data processing

GABARAPL1:SCOC LIR peptide complexes were prepared by mixing purified full length GABARAPL1 and SCOC-2pS peptide (residues 9–19, EED-pS-TFTNI-pS-L) or SCOC-2pT peptide (residues 10–21, EDS-pT-F-pT-NISLAD) at a 1:3 molar ratio. The complexes were dialysed overnight in 25 mM Tris-HCl, pH 8.0, 150 mM NaCl and 0.5 mM TCEP buffer, using a 500–1000 Da MWCO dialysis tubing for both complex. The GABARAPL1:SCOC LIR peptide complexes and the SCOC^6-23^LIR-GABARAP chimera protein were all crystallized at 20 °C using the sitting-drop vapour diffusion method with a protein concentration of 10–20 mg/ml. The initial crystallization trial was performed using Qiagen (JCSG core 1–4, AMSO4), Molecular dimension (PACT, Wizard 1–4), Jena Bioscience (PiPEG). In all cases the drop included 0.5 μl of protein and 0.5 μl of mother liquor. For SCOC^6-23^LIR-GABARAP crystals grew in 50 mM Bicine pH 8.4, 30% PEG 1500. For SCOC-2pS:GABARAPL1 and SCOC-2pT:GABARAPL1 crystals grew in 0.1 M MES pH 6.5, 3.5 M AMSO4, 1% MPD and 0.1 M Tris pH 8.0, 20% PEG 1500, respectively. Crystals were flash-frozen in liquid nitrogen, and X-ray data sets were collected at 100 K on I03, I02 and I04-1 beamline (mx9826-49, mx9826-55 and mx9826-41) of the Diamond Light Source Synchrotron (Oxford, UK). Data collection and refinement statistics are summarized in Table 1. The data sets were indexed and scaled with xia2.^71^ Molecular replacement was achieved by using the atomic coordinates of the peptide-free GABARAP (PDB code: 1GNU) and GABARAPL1 (PDB code: 2R2Q) in PHASER.^72^ Refinement was carried out using Phenix.^73^ Model building was carried out in COOT.^74^ Model validation used PROCHECK^75^; and figures were prepared using the graphics program PYMOL (http://www.pymol.org).

### Bio-layer interferometry assay

Bio-layer interferometry (BLI) is an optical analytical technique for measuring kinetics of interactions in real-time. The biosensor tip surface immobilized with a ligand is incubated with an analyte in solution, resulting in an increase in optical thickness at the biosensor tip and a wavelength shift, which is a direct measure of the change in thickness. Bio-layer interferometry analyses of ATG8s binding to immobilized biotinylated LIR peptides were performed using an Octet Red 96 (ForteBio). 50 μg/ml of biotinylated LIR peptide was immobilized on streptavidin coated biosensor (SA, ForteBio) and the typical immobilization levels were above 0.3 nm. Ligands-loaded SA biosensors were then incubated with different concentrations of ATG8. All binding experiments were performed in solid-black 96-well plates containing 200 μl of solution (25 mM Tris pH 7.5, 150 mM NaCl, 0.5 mM TCEP, 0.1% Tween20, 1 mg/ml BSA) in each well at 25 °C with an agitation speed of 1000 rpm. Each measurement was repeated 2 to 3 times. Dissociation constants for LIR-ATG8 interactions were determined from plotting the increase in BLI response as a function of the protein concentration and fitting using non-linear regression of ForteBio 7.1 data analysis and GraphPad Prism 7 softwares.

The following peptides were synthesized using FMOC solid phase peptide chemistry by the Francis CRICK Institute peptide chemistry science technology platform:

**Table:** 

SCOC WT (aa 6–25):	Biotin-Linker-KEEEEDST**FTNI**SLADDIDH-Amide
SCOC pS12:	Biotin-Linker-KEEEED-pS12-T**FTNI**SLADDIDH-Amide
SCOC pT13:	Biotin-Linker-KEEEEDS-pT13-**FTNI**SLADDIDH-Amide
SCOC pT15:	Biotin-Linker-KEEEEDST**F-pT15-NI**SLADDIDH-Amide
SCOC pS18:	Biotin-Linker-KEEEEDST**FTNI**-pS18-LADDIDH-Amide
SCOC pS12/pT15:	Biotin-Linker-KEEEED-pS12-T**F-pT15-NI**SLADDIDH-Amide
SCOC aa 13–25:	Biotin-Linker-T**FTNI**SLADDIDH-Amide
SCOC aa 11–25:	Biotin-Linker-DST**FTNI**SLADDIDH-Amide
SCOC aa 11–25/pS12:	Biotin-Linker-D-pS12-T**FTNI**SLADDIDH-Amide
SCOC-aa 6–18:	Biotin-Linker-KEEEEDST**FTNI**S-Amide
SCOC aa 6–20:	Biotin-Linker-KEEEEDST**FTNI**SLA-Amide
SCOC aa 6–20/pS18:	Biotin-Linker-KEEEEDST**FTNI**-pS18-LA-Amide

### Immunostaining and confocal microscopy

Cells were grown on coverslips, fixed with 3% paraformaldehyde in PBS for 20 min before permeabilization with methanol at room temperature for 5 min. Coverslips were then blocked in 5% BSA (Roche) in PBS for 20 min. Coverslips were incubated with primary antibody in 1% BSA in PBS 1 hour at room temperature. Coverslips were washed and incubated with secondary antibody in 1% BSA for 1 hour. After final washing with PBS and water, coverslips were mounted in mowiol. Images were acquired using a Zeiss LSM 710 confocal microscope (x63 oil-immersion lens) and Zeiss ZEN imaging software.

### Accession numbers and data availability

The UniProt database numbers (https://www.uniprot.org/) of the SCOC isoforms are: SCOC isoform 1 (Q9UIL1), SCOC isoform 4 (Q9UIL1-4), SCOC isoform 3 (Q9UIL1-3), SCOC isoform 2 (Q9UIL1-2), SCOC isoform 5 (A0A0C4DGB0/protein accession AAK01707). Atomic coordinates and crystallographic structure factors have been deposited in the Protein Data Bank under accession codes 7AA7, 7AA8 and 7AA9. All other data that support the findings of this study are available from the corresponding authors upon request.
