# Corrigendum to Phosphorylation of the LIR Domain of SCOC Modulates ATG8 Binding Affinity and Specificity

**DOI:** 10.1016/j.jmb.2021.167049

**Published:** 2021-07-23

**Authors:** Martina Wirth, Stephane Mouilleron, Wenxin Zhang, Eva Sjøttem, Yakubu Princely Abudu, Ashish Jain, Hallvard Lauritz Olsvik, Jack-Ansgar Bruun, Minoo Razi, Harold B.J. Jefferies, Rebecca Lee, Dhira Joshi, Nicola O'Reilly, Terje Johansen, Sharon A. Tooze

**Affiliations:** 1 Molecular Cell Biology of Autophagy, The Francis Crick Institute, 1 Midland Road, London NW1 1AT, UK; 2 Structural Biology Science Technology Platforms, The Francis Crick Institute, 1 Midland Road, London NW1 1AT, UK; 3 Peptide Chemistry Science Technology Platforms, The Francis Crick Institute, 1 Midland Road, London NW1 1AT, UK; 4 Molecular Cancer Research Group, Department of Medical Biology, University of Tromsø – The Arctic University of Norway, 9037 Tromsø, Norway; 5 Department of Cell and Chemical Biology, Leiden University Medical Center, Einthovenweg 20, 2333 ZC Leiden, the Netherlands; 6 Department of Molecular Cell Biology, Centre for Cancer Biomedicine, Institute for Cancer Research, The Norwegian Radium Hospital, Montebello, N-0379 Oslo, Norway

The affiliation of the corresponding author Sharon A. Tooze to the Institute of Cancer Research, Olso, Norway was incorrectly added. The authors would like to apologize for any inconvenience caused.

