# Supplementary material for: Phosphorylation of the LIR Domain of SCOC Modulates ATG8 Binding Affinity and Specificity
Source: J Mol Biol. 2021 Jun 25;433(13):166987. doi: 10.1016/j.jmb.2021.166987 (PMC8202330; doi:10.1016/j.jmb.2021.166987)
Supplement: Supplementary data 1 [file mmc1.pdf]

Supplementary Information

Phosphorylation of the LIR domain of SCOC modulates ATG8 binding affinity and specificity

M. Wirth and S. Mouilleron et al.

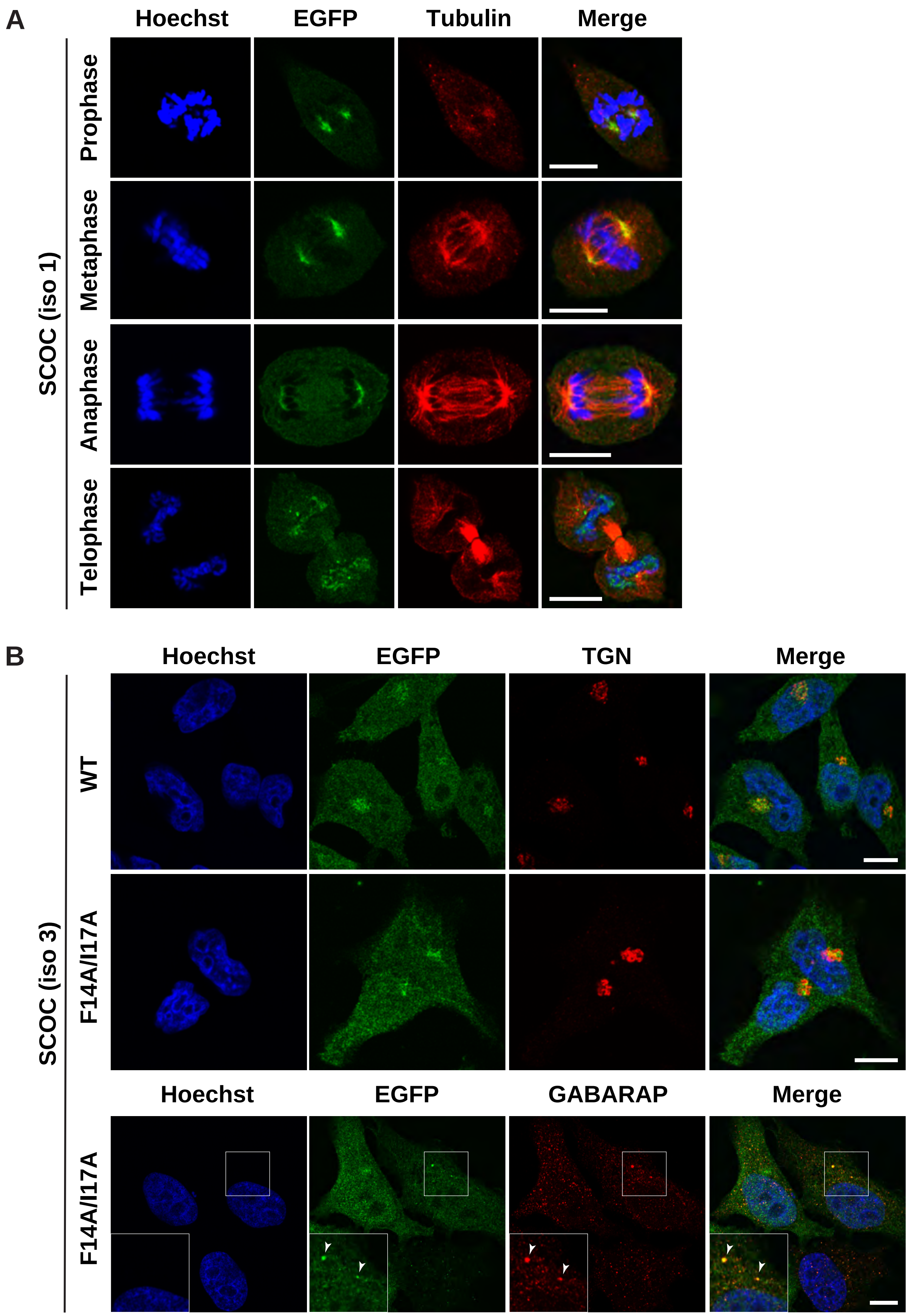

Supplementary Figure 1

Supplementary Figure 1: Subcellular localization of EGFP-SCOC (isoform 1 and 3). A. HeLa Flp-In T-Rex cells (from asynchronous cell culture) stably expressing EGFP-SCOC (isoform 1) starved for 2 h in EBSS in the presence or absence of Bafilomycin A1, fixed and labelled with anti-tubulin and Hoechst for confocal microscopy. **B.** HeLa Flp-In T-Rex cells stably expressing EGFP-SCOC (isoform 3. WT) or EGFP-SCOC (isoform 3, F14A/I17A) starved for 2 h in EBSS, fixed and labelled with anti-TGN46 (upper panels) or anti-GABARAP (lower panel) and Hoechst for confocal microscopy. Expression of EGFP-SCOC constructs was induced with 0.5 μg/ml tetracycline overnight. Scale bars represent 10 μM. White boxes indicate position of insets and arrows indicate EGFP-SCOC F14/I17A (isoform 3)-GABARAP colocalization.

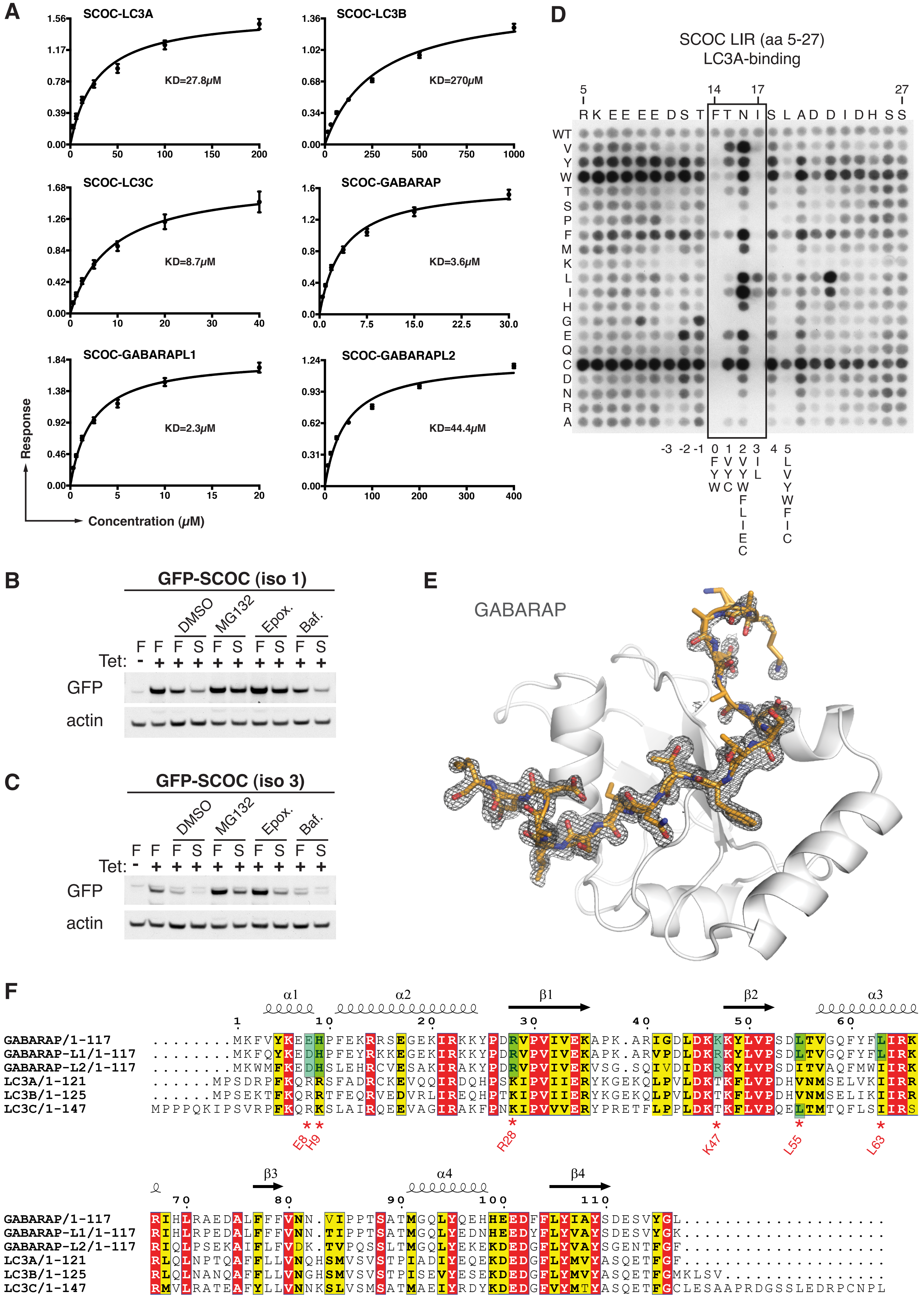

Supplementary Figure 2

Supplementary Figure 2: SCOC binding to mammalian ATG8 proteins. A. Bio-layer interferometry (BLI affinity) measurements showing SCOC WT LIR motif binding to all six human ATG8 proteins. **B.** and **C.** Western blot analyses of EGFP-SCOC isoform 1 (B) and isoform 3 (C) protein levels in HeLa Flp-In T-Rex cells. HeLa Flp-In T-Rex cells stably expressing EGFP-SCOC constructs were grown overnight in full medium supplemented with 0.5 μg/ml tetracycline (Tet) to induce expression of EGFP-SCOC. Tetracycline was removed to shut-off protein expression and cells were treated for 7 h with DMSO, 10 μM MG132, 1 μM epoxymycin (Epox.), 100 nM Bafilomycin A1(Baf.) in full medium (F) or Earle's balanced salt solution (S). Non-treated (-) and Tet-treated (+) cells. **D.** Mutational peptide array of 23-mer SCOC LIR motif and domain incubated with GST-LC3A and immunoblotted with anti-GST. Each amino acid position was substituted for every other amino acid. **E.** Electron density map of SCOC WT LIR (aa 6-23) bound to GABARAP (chimera protein). The Fo-Fc omit map of the LIR motif is contoured at 3.0 σ. GABARAP is displayed in white cartoon and the SCOC LIR domain in orange cartoon and sticks. **F.** Sequence alignment of human ATG8-family orthologs using ESPript [76]. Identical (red) and similar residues (yellow) are boxed. Red asterisks and green boxes indicate non-conserved ATG8 residues involved in binding of wild-type or phosphorylated SCOC LIR motifs to GABARAP or GABARAPL1 based on structural analyses shown in Figures 2D and 5C.

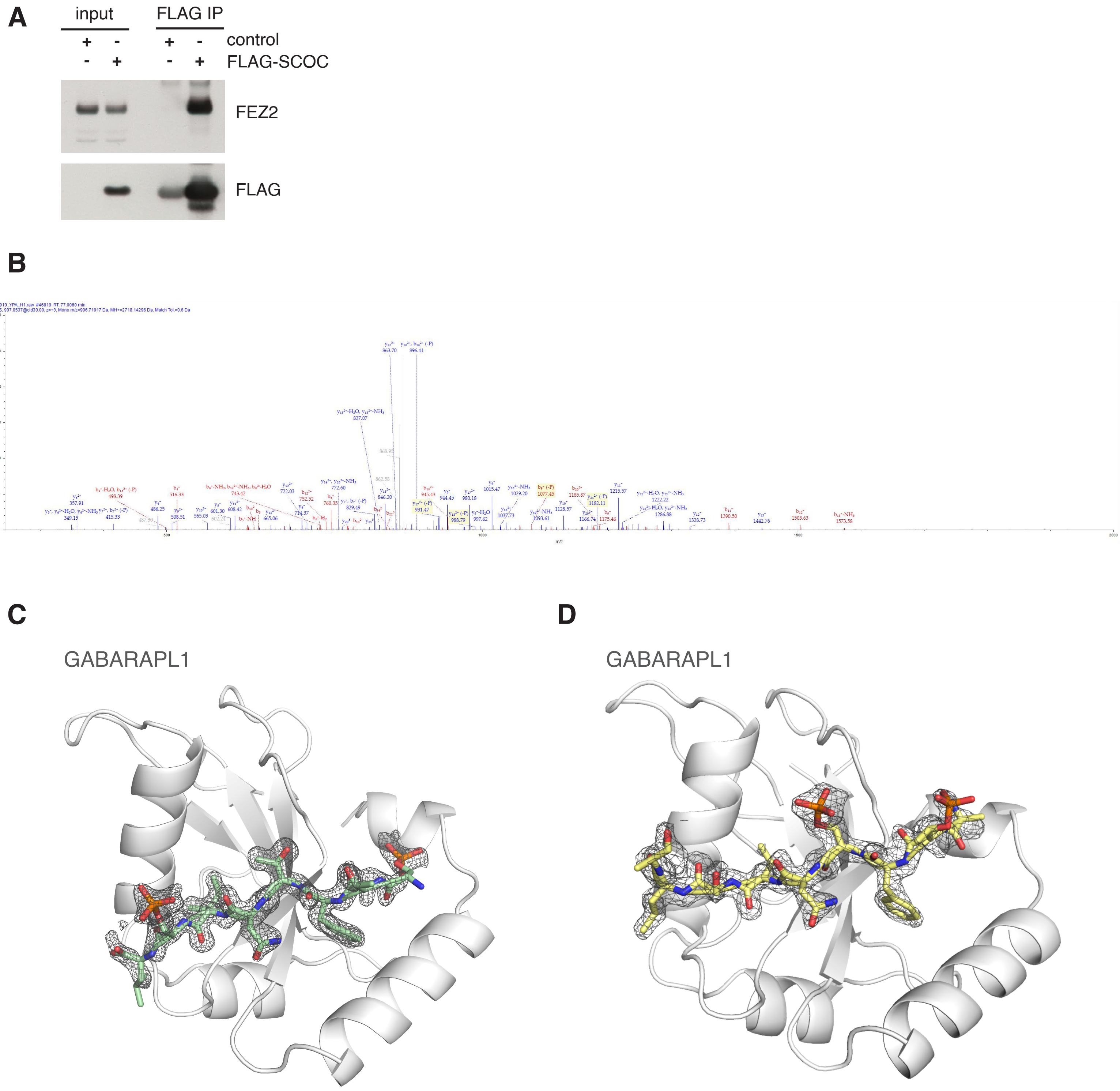

Supplementary Figure 3

Supplementary Figure 3: Binding of phosphorylated SCOC LIR domain to GABARAPL1. **A.** Anti-FLAG immunoprecipitation (IP) of HEK293A cells expressing FLAG-SCOC or control vector (pcDNA3.1) and Western blot with indicated proteins. **B.** Representative MS/MS spectrum for identification of S12 phosphorylation of the KEEEEDSTFTNISLADDIDHSSR peptide using the Proteome Discoverer 2.4 software (ThermoFisher). **C.** and **D.** Electron density maps of SCOC pS12/S18 LIR domain peptide (aa 9-19 shown in green) **(C)** and SCOC pT13/T15 LIR domain peptide (aa 10-21 shown in yellow) **(D)** bound to GABARAPL1. The Fo-Fc omit map of the LIR motif is contoured at 3.0 σ. GABARAPL1 is displayed in white cartoon and the LIR domain in cartoon and sticks.

References

[76] X. Robert, P. Gouet, Deciphering key features in protein structures with the new ENDscript server, Nucleic Acids Res. 42 (2014). https://doi.org/10.1093/nar/gku316.
